# A Comprehensive Review on the Biogenic Amines in Cheeses: Their Origin, Chemical Characteristics, Hazard and Reduction Strategies

**DOI:** 10.3390/foods13162583

**Published:** 2024-08-18

**Authors:** Giuseppe Natrella, Mirco Vacca, Fabio Minervini, Michele Faccia, Maria De Angelis

**Affiliations:** Department of Soil, Plant and Food Science, University of Bari Aldo Moro, Via Amendola 165/a, 70126 Bari, Italy; mirco.vacca@uniba.it (M.V.); fabio.minervini@uniba.it (F.M.); michele.faccia@uniba.it (M.F.); maria.deangelis@uniba.it (M.D.A.)

**Keywords:** cheese safety, biogenic amines, ripened cheeses, soft cheese, tyramine, histamine

## Abstract

Most of the biogenic amines are naturally found in fermented foods as a consequence of amino acid decarboxylation. Their formation is ascribable to microorganisms (starters, contaminants and autochthonous) present in the food matrix. The concentration of these molecules is important for food security reasons, as they are involved in food poisoning illnesses. The most frequent amines found in foods are histamine, putrescine, cadaverine, tyramine, tryptamine, phenylethylamine, spermine and spermidine. One of the most risk-prone foods are cheeses, mostly ripened ones, which could easily accumulate amines due to their peculiar manufacturing process and ripening. Cheeses represent a pivotal food in our diet, providing for nutrients such as amino acids, calcium, vitamins and others; thus, since they are widely consumed, it is important to evaluate the presence of toxic molecules to avoid consumers’ poisoning. This review aimed to gather general information on the role of biogenic amines, their formation, the health issues and the microorganisms and processes that produce/reduce them, with a focus on their content in different types of cheese (from soft to hard cheeses) and the biotic and abiotic factors that influence their formation or reduction and concentration. Finally, a multivariate analysis was performed on the biogenic amine content, derived from data available in the literature, to obtain more information about the factors influencing their presence in cheeses.

## 1. Introduction

Milk and dairy products have been since ever among the most consumed foods worldwide, being part of the ancient culture and tradition of many countries. Historically, cheeses have been essential components of a human balanced diet as well as fundamental foods for population survival, especially in rural areas, where they act as a reservoir for excess milk. The benefits of consuming these products are widely known, since they are vehicles of almost all nutrients (fats, proteins, carbohydrates, minerals and vitamins) while also contributing to the development and maintenance of homeostasis in human gut microbiota [1,2,3,4,5,6].

Unfortunately, beyond these advantages, some drawbacks exist, either from a microbiological or chemical point of view.

Although episodes still occur nowadays even in heat-treated milk/cheese, milk-borne illnesses derived from the consumption of raw milk was widespread until the middle of the 20th century due to the presence of pathogenic microorganisms (i.e., *Escherichia coli*, *Listeria monocytogenes*, *Salmonella* spp., *Staphylococcus aureus*, *Campylobacter* spp., and others) derived from animals or dirty dairy environments [7,8]. To date, outbreaks have been attributed to milk, fresh, soft, or semi-soft cheeses whose high-water content is favorable to pathogen growth causing illnesses such as listeriosis, enterohemorrhagic *E. coli* infection and campylobacteriosis. The last is frequently diagnosed in raw milk consumers, while the salmonellosis hazard mainly concerns powdered infant formula [9,10].

Regarding chemical hazard, many aspects have to be considered, since chemicals can contaminate milk or dairy products in many ways [10]:

(i) Mycotoxins (aflatoxin B1, B2, G1 and G2, ochratoxin-A, fumonisin and others) can contaminate milk from animal feeding. The Aflatoxin M1, which arises from the conversion of aflatoxin B1 by ruminants, is very harmful to human health [11].
(ii) Pesticides arising from contaminated animal feed, although their use has been limited or banned in many countries [12].
(iii) Toxins from plants eaten by animals such as pyrrolizidine alkaloids can represent a risk for consumers, although the carry-over towards milk is very low [13].
(iv) Veterinary drugs, such as antibiotics used for animals affected by mastitis, having the affinity to migrate into the milk. Therefore, those animals being under drug treatment need to have a withdrawal period prior to provide milk eligible for human consumption [14].

Concerning dairy products, the chemical hazard is mostly connected with the following conditions:

(v) Dairy sanitizer and detergents used for cleaning-in-place (CIP) and disinfection of cheesemaking production plants [15] that leave residues when not handled properly; their residues can contaminate the product and consequently pose a risk to consumers.
(vi) Migration of chemicals from packaging (e.g., bisphenol A, phthalates and aluminum) mainly for milk product packaging or bottles used for infant feeding, which may represent a serious issue from childhood to adult health [9,16,17].
(vii) Undesired microbial metabolites, beyond molecules arising from uncontrolled fermentations (i.e., butyric acid, acetic acid, CO_2_ and others, which usually provoke defects leading to economy losses), as well as other molecules that are of safety concern such as biogenic amines (BAs).
(viii) New-borne cheese processing/ripening contaminants originating from chemical reactions among milk constituents, i.e., advanced glycation end products (AGEs), representing a risk for human health [18].

The hazards related to the presence of BAs is the main topic of the present review, whose aim is to describe their origin and chemical characteristics, the role of microbes, the factors enhancing or reducing their formation, and the effects on human body. These molecules are of great interest to producers and regulatory agencies, as they are present in foods (fish and fish products, fruits, nuts, meat products, chocolate) and beverages (fermented beverages, i.e., beers and wines) and can pose a serious health hazard to consumers. Biogenic amines are responsible for several food-borne outbreaks which led to a more conscious consumer that, in turn, asked for safer foods; therefore, researchers over the last few decades focused on biogenic amines identification, characterization and reduction strategies.

The present review focuses on the available literature on soft, semi-hard and hard ripened cheeses, considering all the findings from the late 1980s of the last century until today; the literature search strategy used was based on the Scopus database website by using different queries (i.e., biogenic*amines*cheese*ripening*raw*pasteurization*microorganism and others).

## 2. Origin—Chemical and Hazard Characteristics of Biogenic Amines

### 2.1. How Do They Originate

Amines are classified as endogenous and exogenous. While the former are produced by tissues and exert functional roles in the human body (i.e., dopamine, epinephrine, serotonin, melatonin and others important as neurotransmitters, growth regulation, inflammation mediators), the latter are considered antinutritional factors originated by the decarboxylase activity of fermenting microorganisms in processed foods [19,20]. In fact, the attribute “biogenic” refers to the origin of these molecules, since they are formed by living organisms (i.e., bacteria, molds, yeasts), which decarboxylate the α-carboxyl group of amino acids. In some cases, some BAs originated by a two-step reaction (i.e., decarboxylation and deamination for putrescine formation) or from other amines (i.e., spermidine arises from putrescine by spermidine synthase, or spermine obtained by spermine synthase from spermidine). However, they could also arise by the action of raw material enzymes, and by the amination and transamination of aldehydes or ketones during metabolic processes [21,22]. 

**Figure 1:**
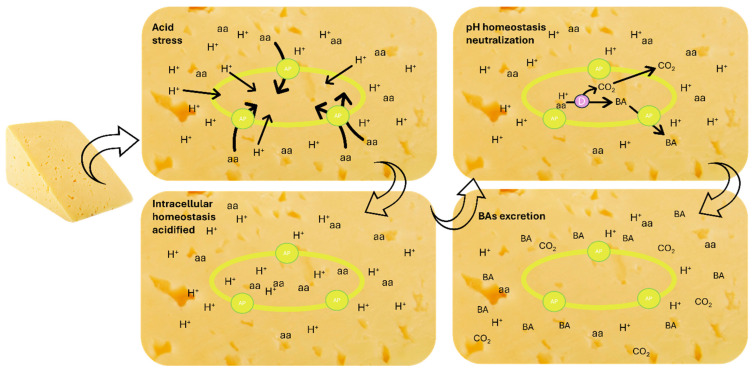
Biogenic amine formation mechanism. H^+^ = weak acid; aa = amino acid, AP = antiporter, D = decarboxylase enzyme, CO_2_ = carbon dioxide, BA = biogenic amine.

The reason why microorganisms synthesize BAs is mainly due to a physiological purpose in response to acid stress and to obtain supplementary energy, reflecting metabolic advantages in adverse ecosystem conditions featuring the matrix. During acidic stress conditions, weak acids and free amino acids (FAA) pass through the bacteria cell membrane and membrane antiporter, respectively, and then the amino acid decarboxylase converts H^+^ and FAA into BAs and CO_2_, useful in restoring the internal pH (Figure 1). In addition, an energetic contribution is excreted by the antiporter and the decarboxylase enzymes, which promotes a proton consumption reaction leading to the generation of the proton motive force, useful for bacteria with no respiratory chain [23].

Certain conditions are needed to produce these molecules, first of all the presence of their precursors. A high amine amount is expected in matrices rich in proteins and where a certain degree of proteolysis occurs, as in the case of ripened cheeses [24,25]. The cheese environment is inclined to BAs accumulation since low pH, low sugars, and hostile environment are extreme conditions for optimal microbial growth, whose metabolism results in amino acid decarboxylation processes to obtain energy, survive and reproduce [26,27]. Landete et al. [28] confirmed this bacterial behavior since reduced production of BAs was observed in vitro as the result of a set of experiments accounting for oenological lactic acid bacteria (LAB) exposed to high sugar content as an energy source. However, there is not always a direct relationship between precursors and their products, because the presence of the former is not the unique factor influencing BA production. Thus, the BAs final concentration in foods is the result of a combination of many important factors, such as food processing conditions (processing temperatures, time, raw matter chemical characteristics, storage) and microorganisms (BAs-producing and BAs-consuming) [29]. Concerning cheese, to date there is no absolute rule in predicting the BAs content; however, the evaluation of all the factors involved in cheese manufacturing could help provide a clue on the degree of contamination. For example, all of the parameters cited above together play an important role in determining the BAs accumulation, i.e., ripening time favors the release of precursors, the use of raw matter could influence the presence of specific bacteria with decarboxylase activity, the type of cheesemaking, ripening environment, and handling of the product could influence the growth of specific microflora, leading to a more or less contaminated product, etc. Thus, for example, theoretically fresh cheeses will contain less amines than ripened ones, as well as raw milk cheeses will have higher BAs than pasteurized milk cheeses. Albeit sometimes it reflects the real situation (i.e., fresh vs. ripened cheeses), this is not always true and there is a need to look at the overall picture.

### 2.2. Chemical Structure and Consumers’ Toxicological Issues

The BAs can be additionally classified by their chemical structure (aliphatic, aromatic, heterocyclic) or number of amine groups (monoamine, diamines, polyamines). Among all of these, histamine, tryptamine, putrescine, cadaverine, phenylethylamine (or 2-phenylethylamine), tyramine, spermidine and spermine are the most prevalent amines detected in foods (Table 1) [20,30]. According to some authors, spermine and spermidine are no more considered “true” biogenic amines, due to their origin (not directly arising from amino acid) and due to their low concentrations found in foods, which determines their non-related health issues, although health problems have been attributed at high concentrations [31,32].

Notably, most of BAs take part in some body regulation activities, i.e., histamine plays an important role in cerebral activities, regulation of the body temperature and stomach volume, and it also has a role in starting allergic reactions, whereas tyramine increases the blood pressure as a vasoconstrictor. Thus, considering their important roles in human physiology, a detailed study on this topic was performed by Erdag et al. [22].

To prevent unhealthy events, the BAs concentration in the human body is regulated by a defense mechanism consisting of BA detoxification (both endogenous and exogenous) by oxidation enzymes (i.e., diamine oxidase, polyamine oxidase and monoamine oxidase). However, according to Ruiz-Capillas et al. [35], the defense mechanism is not always effective, as many factors can influence its activity, such as drugs (analgesics, antidepressants and others), immune deficiencies, gastric dysfunction, or alcohol consumption. In addition, putrescine and cadaverine can potentiate histamine and tyramine toxicity, leading to potential adverse effects even in healthy consumers. 

Thus, an excessive intake of BAs can cause adverse events even in a healthy consumer due to insufficient amine metabolism; however, in people with health issues (children, elderly, pregnant women and others), where the oxidative activity could be weaker, the toxicological effect could be amplified [36]. In general, the ingestion of high amine concentrations basically represents a food hazard risk. More information is available by consulting the in-depth section in the Supplementary Materials (Deepening S1).

### 2.3. What Produces Amines in Cheeses

Among the living organisms in cheese, bacteria are mainly responsible for BAs production due to their abundance, activity and presence of the decarboxylase enzyme in their genome. In line with this, the highest concentration of BAs was found as the result of relevant microbial density (>7 Log CFU g^−1^). Among cheeses, ripened ones are characterized by long fermenting processes with a succession of microorganisms from starter cultures (SLAB), secondary cultures, NSLAB, contaminants and wild strains. The more complex microbiota is, the greater the probability for the presence of BAs-producing species, and the presence of a complex and heterogeneous microbiota is also affected by the raw matter quality and heat treatments applied (raw, pasteurized or thermized milk). Also, the cheese ripening time influences the BAs accumulation compared to fresh cheeses due to free amino acids release during the proteolysis process [37]. However, reducing the related precursors does not represent a valid alternative to mitigate BAs accumulation, as amino acids are essential for the proper chemical and sensory evolution of ripened cheeses [38].

Furthermore, microbial load is an important factor. Although it should be considered that not all microorganisms are BAs producers, both Gram-positive and Gram-negative bacteria can possess the decarboxylases gene [36], including pro-technological bacteria (LAB, yeasts and molds). In light of this view, a holistic approach should consider environmental conditions (i.e., ripening environment and temperature) as well as microbial species and strains [33,39].

The microbial species involved in the BAs production in dairy products are different, encompassing both spoilage and pre-technological bacteria, as discussed in the work carried out by Benkerroum [33] that reported an exhaustive list of BAs-producing bacteria species of dairy relevance. Additionally, it is worth noting how a microorganism’s decarboxylase activity, in some cases, is not highly specific towards a single amino acid, as it represents a sort of emergency mechanism in response to a stress. In fact, it was stated that microorganisms are able to convert amino acids with similar structure in the absence of the primary amino acid: i.e., tyrosine decarboxylase, which usually converts tyrosine to tyramine, is also able to convert phenylalanine to phenylethylamine [40]; ornithine decarboxylases convert ornithine to putrescine but also lysine to cadaverine [41]; arginine decarboxylases also work with ornithine [42]. The activity of these enzymes is not related to cell viability; in fact, their activity was also observed after cell damage.

Tyramine is the main amine found in dairy products responsible for the so-called “cheese effect”, and it is mainly produced by Gram-positive bacteria such as lactobacilli (*Lactobacillus brevis*, *reuteri*, *casei*), lactococci (*Lactococcus lactis* subsp. *lactis* or *cremoris*), enterococci (*Enterococcus faecalis*, *faecium*, *durans*), as well as others. Among Gram-negative bacteria, *Pseudomonas*, *Citrobacter*, *E. coli* and others certainly represent the main taxa featuring the milk and cheese microbiota that are responsible for the synthesis of BAs [11,33,39,40,43]. According to many researchers, the presence of high densities of *Enterococcus* spp. in milk pivotally influences the tyramine content in cheese [44,45]. Concerning the second most important BA, histamine, this can be produced mostly by Gram-positive and also by Gram-negative bacteria. Furthermore, concerning Gram-positive bacteria, it possible to mention *Streptococcus thermophilus*, lactobacilli (*Lactobacillus parabuchneri*, *buchneri*, *curvatus*, *helveticus*, *sakei*), *Clostridium perfringens* and *Staphylococcus xylosus*. By contrast, *Klebsiella* (*pneumonia*, *oxytoca*), *Enterobacter* (*aerogenes*, *gergoviae*) and *Pseudomonas putrefaciens* are the most representative within Gram-negative bacteria.

As it is possible to observe, most of these histamine- and tyramine-producers are SLAB used for cheesemaking; however, a synergic BAs-producing effect was observed between *S. thermophilus* and lactotocci, despite both are claimed as safe by EFSA and widely employed in the cheesemaking process [46]. Thus, it could be hypothetically useful to search for additional starter cultures without decarboxylase genes [43,47]. Furthermore, it should be emphasized that when using raw milk in cheesemaking, it is plausible to find autochthonous bacteria, likely LAB, possessing decarboxylase activity and, therefore, contributing to enriching the matrix with pleasant compounds during ripening that, at the same time, could contribute to biogenic amine production and accumulation [48,49]. Considering this evidence, many traditional cheeses made with raw milks might have, on average, a higher BAs content than pasteurized milk-based products [50]. The heat treatment improves the microbial quality of the milk, reducing the total count and specific spoilage bacteria (e.g., *Enterobacteriaceae*) resulting in a production with reduced content of the bacteria implicated in BAs production [51]. To corroborate this hypothesis, results on goat milk provided by Novella-Rodríguez et al. [52] demonstrated reduced total microbial and *Enterobacteriaceae* counts in pasteurized compared to raw milks, leading to the assessment in respective cheeses of a different BAs content, with cheese obtained from pasteurized milk containing significantly lower amine concentrations. Similar results were confirmed by Schirone et al. [53] on sheep milk cheese. The LAB ability to decarboxylase amino acids arises through horizontal encoding acquisition (plasmids) from other species [54], or is a species-level characteristic as for *E. faecalis*, *faecium* and *durans* [55]. Cadaverine and putrescine are widely produced by the *Enterobacteriaceae* and *Pseudomonadaceae* families or LAB [30,56,57]. On the other hand, the ability to produce BAs could not be considered always at species-level, as some strains might have lost the ability to produce BAs due to a reductive genome evolution, as for some specific *L. Lactis* strains towards putrescine production [58].

Additionally, yeasts (i.e., *Geotrichum candidum*, *Debaryomyces hansenii* and *Yarrowia lipolytica* extracted from cheese) also demonstrated their contribution to decarboxylate ornithine, tyrosine, lysine and histidine [59,60].

### 2.4. Factors Influencing BAs Accumulation—Organization of Case Studies

BAs were found also in fresh cheeses, kefir, and yoghurt, despite their presence is still unclear as these products are characterized by short processing times and limited shelf-life. Therefore, it was hypothesized that their presence could be accountable to endogenous pathways or microbial activities [61,62,63]. Nonetheless, BAs in fresh cheeses is far too low when compared to ripened cheeses, since BAs production pivotally involves proteolysis [64]. In fact, various research reports highlight the correlation between BAs and long-ripened cheeses [65,66,67,68,69,70]. However, cheese ripening is a relative concept because ripening means chemical maturation of the cheese, which does not necessarily require long times, while it is also influenced by consumer preferences; some may prefer a less ripened product while others may prefer a more ripened one. Apart from these considerations, the ripening period, which varies depending on the type of cheese, can range from a few weeks to one, two or more years [71] and, therefore, cannot be considered as a unique statement but deeply defined and contextualized.

Other influencing factors can also be considered in BAs accumulation; reports in the literature also evaluate the ripening conditions and ripening environment, and the presence of specific starter cultures, autochthonous microorganisms, cheese modifications and other factors. This review is organized considering separately hard and semi-hard cheeses from soft cheeses, and further divided by milk breed species, aimed to provide a new point of view in evaluating the amine formation and accumulation in cheeses. The reason milk breed origin was chosen is due to different chemical characteristics featuring different milks, where major and minor nutritional components and the microbiota are known to vary from species to species [72,73].

Concerning one of the major components of milk, proteins, result in a range of 3–3.9% in cattle, 3–5.2% in goat and 4.5–7% in sheep [72]. Consequently, the higher the protein, the higher the possibility to have BAs precursors; despite this, it is not enough because a certain microbial density leading to protein breakage is needed. Concerning this, raw milk microbiota varies greatly according to the legal limit imposed by EU Regulation 853/2004 [74]. In bovine milk, total microbial count (TMC) has to be lower than or equal to 5 Logs UFC/mL; whereas, in small ruminants (ovine and caprine milks), the admissible density is raised 15-fold, and differences in TMC greatly affect cheesemaking when obtained from milk that has not been heat treated. Even somatic cell count (SCC) must be lower than or equal to 400,000 cells per mL for bovine milk, whereas no legal limit is imposed for small ruminants. Due to the absence of a unique limit, Corrales et al. [75] suggested 1,500,000 cells per mL as maximum admissible, whereas other researchers tried to introduce a classification including both goat and ewe bulk milk as “good quality”, with SCC < 750,000 cells; “intermediate quality”, 750,000 < SCC < 1,500,000 cells per mL; and “bad quality”, exceeding 1,500,000 cells per mL [76,77].

Thus, it is clear that the suggested parameters could discriminate bovine from goat and ewe raw matters, as normally non-infected small ruminants showed a higher SCC level [78,79,80]. The SCC considers different animal cells (i.e., macrophages, lymphocytes, polymorphonuclear neutrophils, epithelial cells, cell fragments and cytoplasmic vesicles), and this parameter is generally used as an indicator of milk quality and udder state of the animal’s health status [81], which is important for their impact on the quality and safety concerns of the final milk-based products. In fact, literature reports include different studies in which cheese properties were negatively affected by high SCC levels [82,83,84,85,86]. Additionally, a detrimental enzymatic effect on proteins could be promoted by milk indigenous enzymes (plasmin, elastase, cathepsin D), which could arise from plasma or high SCC [87]. The activation of the endogenous plasminogen to plasmin system, which is a heat-stable protease, can contribute to the first cheese proteolysis process during ripening. Even macrophages and polymorphonuclear neutrophils can contribute to produce other protease as lysosomal enzymes and elastase [88,89,90]. Finally, the SCC lysis releases into the matrix numerous enzymes, including proteases such as collagenase, elastase, cathepsins B, C, D and G [91,92]. Summarizing, most of the available literature supports the positive correlation occurring between high SCC and proteolysis [87,93,94,95,96,97,98], which could theoretically lead to higher BAs precursors. Nonetheless, to the best of our knowledge, Ubaldo et al. [97] is the only research group that investigated and correlated values of BAs with SCC and observed a concrete concentration of tyramine and tryptamine in mozzarella samples produced with high SCC milk.

Only few examples of differences between cheeses made using milks from different animal species are discussed above; thus, together with type of rennet, starter cultures and others, this should be considered as an additional factor that can widely influence the BAs concentration in cheese.

## 3. Hard and Semi-Hard Cheeses—Case Studies

### 3.1. Cow’s Milk Cheeses

Innocente & D’agostin [99] evaluated the BAs concentration in Montasio cheese considering the ripening, from 60 to 150 days, and the proteolysis rate. A correlation was observed, the higher the former, the higher the latter (R^2^ = 0.9827), despite this relation followed a non-linear trend but a second-order polynomial equation, as the release of amino acids occurs during secondary proteolysis during ripening [100]. However, the total concentrations reached an average of 860 mg/kg after 150 days of ripening, in which histamine, tyramine and putrescine were the most abundant compounds. 

Arlorio et al. [65] examined proteolysis in Toma Piemontese PDO cheese during ripening, assessing changes in nitrogenous compounds and BAs. Long-ripened cheese had higher proteolysis levels (after 64 days), with histamine and tyramine detected up to 145 mg/kg. Commercial samples showed total BAs concentrations below 219 mg/kg. Compared to short-ripened Toma, long-ripened cheese displayed more significant proteolysis, including the disappearance of the αs1-Casein band in electrophoresis profiles.

Campos-Góngora et al. [101] correlated the histamine and tyramine concentrations in eight different brands of Chihuahua cheeses with the presence of their respective decarboxylase enzymes, finding a positive correlation among them. Concerning the amines concentration found, 3 out of 8 samples showed the presence of histamine (46–192 mg/kg), while tyramine was found in 6 out of 8 samples at higher concentrations (115–209 mg/kg). The latter do not pose a risk to consumers; on the other hand, histamine concentrations were above the NOAEL suggested by EFSA, representing a possible hazard to consumers. The authors concluded that the use of good manufacturing hygiene practices and higher raw milk quality should reduce BAs content in cheese. 

The influence of unconventional cheesemaking on BAs concentration was also studied. Innocente et al. [102] evaluated the peculiar technology of processing of Asino cheese as an influencing factor of BAs. Traditionally, Asino cheese undergoes a distinctive soaking phase where it is immersed in a special brine called “salmuerie” followed by further ripening. In contrast, the control cheese was continuously ripened in a standard warehouse setting. The results showed higher total BAs in traditional Asino cheese (soaked one) than the control (2079.42 vs. 1635.51 mg/kg), and, therefore, the authors concluded that the presence of BAs in the Asino cheese could be attributed to the diffusion from the salmuerie, where elevated accumulation of BAs was observed. Similarly, Decadt et al. [103] have identified in brine of some defective Gouda cheeses one of the main sources of BAs-producing bacteria, causing presence of putrescine and cadaverine which, in turn, could be directly responsible for off-flavor and indirectly for structural defects (cracks) by late accumulation of CO_2_ in the firmer cheese core. Still concerning Gouda cheese, Kim et al. [104] treated it with Hwangto coating, which contains grapefruit extract, to control the growth of fungi and bacteria during ripening. Results showed differences between the coated and uncoated samples, with the former having a significantly lower content of BAs.

In general, authors have found positive correlation between ripening time and amine content, and decarboxylase enzymes and some amine found in cheeses. The total concentration varies from few mg per kg to about 1000 mg/kg, except for Asino cheese, in which a total of about 2000 mg/kg was found, representing a hazard for consumers.

### 3.2. Ewe’s Milk Cheeses

Concerning the study of BAs in cheeses made from ewe’s milk, various papers are present in the literature, and most of them considered ripening time, ripening environment, milk thermal treatment, presence of indigenous starters, the use of hurdle technology and animals’ diet as influencing variables.

Ordóñez et al. [105] considered as variables the milk heat treatment (raw, pasteurized), the use of commercial starters (*Lactococcus lactis*, *Lactococcus cremoris*) and indigenous bacteria cultures (Type A: *Lactococcus lactis* subsp. *diacetylactis* GC57 and *Lactobacillus casei* subsp. *casei* GC59; Type B: *Lactococcus lactis* subsp. *diacetylactis* GC58 and *Lactobacillus casei* subsp. *casei* GC59). They concluded that pasteurized milk cheeses showed reduced BAs, whereas commercial starters showed a higher content of tyramine and putrescine with a lower amount of histamine, cadaverine, tryptamine and others compared to indigenous starter cultures.

A similar study was conducted by Martuscelli et al. [106], which assessed BAs in ripened Pecorino Abruzzese cheese made from raw milk without starter and from pasteurized milk added with SLAB (*Streptococcus thermophilus*, *Lacticaseibacillus casei* and *L. delbruekii* subsp. *bulgaricus*). The former showed high concentrations of histamine and tyramine (approximately 280 and 190 mg/kg, respectively) in ripened cheese, whereas phenylethylamine, tyramine, and putrescine (approximately 300, 280 and 190 mg/kg, respectively) mainly featured the latter. Considering the ripening, the highest amounts of histamine differed between cheeses, as those made with raw milk reached the highest concentration after 60 days of ripening, while those made from pasteurized milk and SLAB reached the highest concentration after 30 days, although a decreasing trend was then observed.

Combarros-Fuertes et al. [107] considered how milk heat treatment, ripening time, and different ewe milk breeds influenced the content of BAs in Zamorano PDO cheese. Results showed that both ripening time and absence of milk heat treatment significantly affected the BAs content. In fact, long ripening time and raw milk sample cheeses showed total higher amine content than long ripening time and heat-treated milk cheeses (about 600 vs. 200 mg/kg, respectively). By contrast, the use of different milk breeds did not affect the BAs amount. 

Similar results were found by Andic et al. [108] on a typical herby cheese made from ewe milk found high BAs in conjunction with long ripening time. Among 30 samples, 10 were heavily contaminated (up to about 5000 mg/kg).

The importance of the ripening environment on BAs production was studied by Torracca et al. [109,110], who analyzed four different cheeses, three out of four made with raw milk and the fourth from pasteurized milk, all ripened in controlled rooms: “grotta”, “fossa” and tuff cave. The authors, contrary to what had been reported before, concluded that milk pasteurization did not affect the BAs content. By contrast, ripening conditions deeply influenced the BAs production, since cheeses ripened in a controlled room had the lowest values (also due to the shorter ripening period, about 200 mg/kg), while cheeses ripened in uncontrolled hygiene conditions reached a very high BAs concentration (i.e., “fossa” and tuff cave ripening had >1000 mg/kg). Similar results were provided by Mascaro et al. [111], who found higher BAs concentrations in Formaggio di Fossa when ripened in the typical environment compared to the same cheese ripened in a factory (>2500 vs. <100 mg/kg, respectively).

A similar study inspecting the role of the environment was conducted by Schirone et al. [112] on Pecorino di Farindola cheese. The results on 90-day ripened cheeses (10 samples from 10 different dairy farms) revealed a great variability in BAs content (from 209 to 2393 mg/kg), and this was attributed to the different hygienic conditions of the producers. Proteolysis extent plays an important role in BAs formation, and the authors hypothesized that its great contribution is accountable to the proteolytic enzymes of the pig rennet used.

The effect of cheesemaking season on chemical and sensory profile was considered by Renes et al. [113] on a Pecorino cheese made from pasteurized ewe milk. Summer cheese was the richest in BAs, probably due to the fact that raw milk microbiota reached higher total microbial counts and were resistant to pasteurization (total BAs of 740 and 910 mg/kg after 100 and 180 days of ripening, respectively). After 100 days of ripening, cadaverine, tyramine and histamine were the most abundant BAs; moreover, tryptamine was found only in summer and spring cheeses (124 mg/kg after 100 days of ripening in summer vs. 2 mg/kg after 180 days of ripening in spring). In view of these results, which were also corroborated by Joosten [114], BAs content depends on amino acids precursors, which in turn are related to the action of the indigenous and exogenous proteolytic enzymes, all factors that are significantly influenced by environmental factors (i.e., pH and temperatures).

Pintado et al. [115] studied 29 samples of Terrincho, a Portuguese cheese, aiming to define a correlation between the microbiological–chemical profile and the BAs content. Results highlighted a qualitative and quantitative positive correlation between microorganisms and BAs. In detail, enterococci correlated with phenylethylamine, lactococci with cadaverine and tyramine, and enterobacteria with tryptamine and cystamine. From a sensory–chemical point of view, low pH and small amounts of salt favored the production of BAs. Although the samples were taken in the same PDO region, the hygiene conditions of the dairies greatly influenced the amine accumulation in the products. The improper handling of raw milk could contribute to an enrichment of bacteria producing and releasing decarboxylase enzymes into the matrix, thus, contributing to biogenic amine formation into the product even in the absence of viable cells [112]. In addition, the total BAs was quite high, varying from about 400 to about 1000 mg/kg.

Also, new advances in this research field have been considered to control autochthonous bacteria in cheeses obtained from raw milk. Calzada et al. [116] applied a high-pressure treatment (400 and 600 MPa) to cheese, and the results showed a reduced microbial count that, in turn, reduced the decarboxylase enzyme activity and BAs content. Another study evaluated the effect of a diet containing 10% grape pomace on the quality of dairy products during ripening, revealing how this diet modified the cheese microbiota and increased the diamines content [117].

The content of amines in ewe milk cheeses reflects higher amine variability; in fact, the literature reports samples with low amine content (about 100 mg/kg) up to several thousand per kg. In contrast to cow milk cheeses, it is difficult to define a specific factor influencing their accumulation, as the literature sometimes is controversial. In some cases, milk heat treatment could be useful, in other cases not, probably because it depends also on the cheese ripening environment, by the milk microbial quality and the hygiene of the dairy and materials.

### 3.3. Goat’s Milk Cheeses

Concerning cheese made from goat milk, few papers were available in the literature. Galgano et al. [118] tried to find a relationship between BAs and enterococci in fresh and ripened goat cheese. After 60 days of ripening, a total of 1313 mg/kg of BAs was found, with putrescine as the most abundant. This was attributed to low hygienic conditions during the cheesemaking process and to a high number of coliforms. The hygienic conditions and microbial loads were also evaluated by Novella-Rodríguez et al. [52] in different goat cheeses obtained from raw and pasteurized milks. As for the previous study, enterococci were found to be mainly responsible for tyramine and phenylethylamine production, along with lactobacilli in raw milk cheese. Moreover, higher amounts of *Enterobacteriaceae* were found in raw milk cheese than the pasteurized one. The former had a higher BAs content than the latter since heat treatment reduced the load of BAs-producing bacteria. In this case, both ripening time and milk heat treatment influenced the amine concentration; considering the total amounts at the end of the period monitored (90 days), the pasteurized milk cheese had about 100 mg/kg against about seven times more for the raw milk cheese. 

Poveda et al. [119] found no correlation between the BAs precursors (free amino acids) and BAs alone in a pasteurized goat milk cheese. In addition, many authors have tested the ability of bacteria possessing the histidine decarboxylase gene to produce histidine and have shown no amine formation. This suggests that bacteria do not always produce BAs in vitro, whereas in cheese, bacteria find a mix of factors during ripening (pH, temperature, time, etc.) leading to the BAs production [60,120,121].

There are fewer papers concerning the evaluation of goat-milk cheesemaking parameters on BAs accumulation. However, as for ewe milk cheese, high amine concentration variability was found.

### 3.4. Mixed Milk Cheeses

Many cheeses are made from pure cow, ewe, or goat milk, whereas others are a mixture of different milks (i.e., goat and cow, ewe and goat, etc.). Pachlová et al. [122] evaluated the BAs content of cheeses made from different mixtures of goat and cow’s milk (100:0 goat–cow, 75:25, 50:50, 25:75 and 0:100). Differences in proteolysis rate and BAs content were observed among samples, as the most intense proteolysis was found where goat milk was predominant, whereas an opposite trend was observed for cheeses with a higher quantity of cow’s milk. Concerning BAs content, a higher concentration was found in cheeses with a higher amount of goat milk, reaching values above 100 mg/kg. Schirone et al. [53] investigated 12 commercial Pecorino cheeses obtained from either pure ewe milk or a mixture of cow and ewe’s milk. The study aimed to quantify BAs and the bacteria responsible for their production. Remarkably high concentrations of BAs were found, with concentrations reaching up to 5861 mg/kg in Pecorino di Fossa, a cheese made from a blend of cow and ewe milk ripened in a natural pit (“fossa”), an environment lacking control. This cheese exhibited a notably high count of BAs-producing bacteria (10^7^ CFU g^−1^). Additionally, other two Pecorino cheeses ripened in uncontrolled environments—a natural cave (“grotta”) and under-hay (“sotto fieno”)—showed high content of tyramine and histamine despite these were obtained from pasteurized milk. Overall, all samples had significant BAs content, posing potential risks, particularly for individuals using monoamine oxidase inhibitor drugs.

## 4. Soft-Ripened Cheeses—Case Studies

### 4.1. Cow’s Milk Blue Cheeses

Blue cheeses are peculiar products in which molds (i.e., *P. roqueforti*) grow in the inner and outer parts of the cheese, providing its typical appearance and flavor [123]. The literature reports different studies focusing on BAs, as well as innovative strategies enabling the control of their formation. Rabie et al. [124] treated blue cheeses with γ-irradiation at different doses (2, 4, 6 kGy) and confirmed how the γ-irradiation treatment effectively reduced the microbial load while possibly destroying BAs. In fact, while total amine content increased from 1000 mg/kg at day 0 to above 2300 at day 90; the γ-treated samples showed lower concentrations (except for day 0), reflecting the irradiation intensity treatment. Similar results were also found by Kim et al. [125], who found a radiolytic degradation of amine by using gamma irradiation in a model system

Another study specifically focused on six BAs by searching in 46 blue-veined cheeses to integrate the risk assessment data for consumers [126]. The total BAs amount ranged between 5.5 and 824 mg/kg, and almost a quarter of the total sampling had a total concentration below 10 mg/kg. Overall, the authors stated that the BAs variability was widely influenced by the different cheesemaking processes and geographical origins.

StandaroVá et al. [127] evaluated how the BAs content was affected by different factors (i.e., producer, batch, storage time, BAs distribution in cheese) in 66 samples of Niva blue-veined cheese. The total BAs content ranged between 38 and 986 mg/kg, with cadaverine and tyramine the most abundant molecules found, with average values of 188 and 78 mg/kg, respectively. Concerning the batch factor, differences were found to be related to different production months with the June batch having the highest amount of cadaverine and total BAs content, whereas September had the lowest concentration of BAs, with normal exposure to different contamination levels. The BAs content was also evaluated by comparing the core and the edges of the cheeses, providing evidence on how the core always reported higher values than edges without differences among cheese producers.

Gurkan et al. [128] studied the influence of ripening temperatures (4 and 10 °C) and three different strains of *P. roqueforti* on BAs formation in mold-ripened Civil cheese during 90 days of ripening. Higher ripening temperatures and longer ripening time led to a greater BAs production. After 90 days, in cheeses ripened at 10 °C, total BAs ranged from 14 to 45 times higher than cheeses ripened at 4 °C. In addition, two out of three *P. roqueforti* strains tested were found to be particularly prone to BAs production. 

Diezhandino et al. [129] evidenced differences in BAs content in blue cheeses made with raw or pasteurized milk, with the latter having a more intense proteolysis with higher free amino acids concentration but lower BAs with respect to the raw milk cheese.

As widely reported in other studies, the influence of storage time was again demonstrated by Qureshi et al. [130], who investigated the BAs formation during the ripening (around 90 days) of Gamalost cheese, a typical Norway mold-ripened cheese, which could be considered as hard-type but it is also considered as blue cheese. This product is peculiar since it is produced from skimmed milk, which allows for high protein content. Furthermore, it is featured by extensive proteolysis due to the presence of *Mucor mucedo*, a mold species with a high proteolysis activity. Although high BAs content is expected, only putrescine was detected in 20- to 30-day ripened cheese, reaching the threshold of 25 mg/kg that decreased to 16 mg/kg until the 60th day of ripening. Based on these results, the authors noted the scarce decarboxylase activity of *M. mucedo*.

In this category of cheeses, is possible to find cheeses with very low amines content, such as Gamalost, and others that are very contaminated (up to 2300 mg/kg). Once again, it could depend on raw matter quality and dairy hygiene, but in this case, the decarboxylase activity of molds is most important, which is of pivotal importance in cheesemaking.

### 4.2. Cow’s Milk Smear-Ripened Cheeses

Smear-ripened cheeses are products designed to obtain a surface covered by bacteria, resulting in a viscous or red-orange-colored rind. The other name attributed to this type of cheese is washed-rind cheese, since dairy operators used to wash the rind during ripening several times by using a brine [131].

Dugat-Bony et al. [132] studied the dynamic microbial and BAs evolution of a reduced sodium chloride surface-ripened cheese, revealing the importance of the NaCl for the optimal cheese ripening, as the lower NaCl content led to various cheese modifications. In detail, a different microbial development (more spoilage bacteria), changes in proteolysis rate (greater primary and secondary proteolysis), and BAs formation (higher level of some amines, as cadaverine 460 vs. 335 mg/kg in reduced-salt and control cheese, respectively) was found.

Cwiková & Franke [120] revealed that storage temperature influences the BAs content of smear-ripened cheeses, since refrigerated storage showed the highest BAs production compared to freezing temperature storage; hence, providing evidence on how refrigerated conditions slowed enzyme activity but did not stop it. Also, the time of storage had a greater influence on BAs, particularly of tyramine, putrescine, spermidine and spermine concentrations.

The study designed by Komprda et al. [133] was similar to that of StandaroVá et al. [127] on Niva blue-veined cheeses, but researched the impact of different factors (production season, cheese shape, storage temperature and ripening time) on BAs in smear-ripened cheese (Olomouc curd cheese). Storage time and temperature were defined as the main factors affecting BAs in cheese and, notably, the higher the storage temperature, the higher the BAs concentration (about 1000 vs. about 4000 mg/kg, respectively). Also, the cheese shape influenced the BAs, where disc-shaped cheese had on average a 25% higher content than bar-shaped cheese. Contrary to that reported by StandaroVá et al. [127], the results provided by Komprda et al. [133] indicate no differences based on seasonality.

### 4.3. Cow’s Milk Surface Mold–Ripened Cheeses

As reported by Spinnler [134], surface mold-ripened cheeses are those characterized by a felt-like coating composed of white mycelia as the result of mold growth (i.e., *Penicillium camemberti*) on the surface. The mold growth gave rise to cheeses with a typical appearance and unique sensory properties, which make them very popular among consumers.

Metzler et al. [135] and Mackù et al. [136] studied the influence of different molds with different proteolytic activity and ripening time on the BAs content in Camembert cheese, respectively. Both studies concluded that Camembert has a disadvantageous environment for BAs production, as low BAs content featured in all products (<100 mg/kg).

### 4.4. Others

Following are the publications concerning soft cheeses without inner or outer molds and obtained by mixture of different breed milks. Among these, Ricotta Forte is a traditional Italian cheese made from sheep, cow, goat, or a mixture of these, characterized by a prolonged shelf-life with intense proteolysis and lipolysis, favoring the BAs production. In this regard, Rea et al. [137] evaluated the presence of BAs concentration in three different Ricotta Forte cheeses and found very high amounts of cadaverine, putrescine and tyramine, with total BAs ranging 2089.98–10,294.01 mg/kg for the products analyzed. However, Ricotta Forte is not consumed as is, because very small amounts are used as a flavoring ingredient in recipes and, therefore, the BAs intake is very limited.

Valsamaki et al. [138] evaluated the BAs content during ripening of Feta cheese made from 70% ewe milk and 30% goat milk. They concluded that during the first 15 days and at 120 days of ripening the BAs production is more intense, probably due to the high ripening temperature during the early days and the high abundance of precursors in late ripening. Also, Ma et al. [139] evaluated biogenic amine content in different commercial cheeses, including Feta. They found total concentrations ascribable to that found by Valsamaki et al. [138] after 15 days of ripening (about 250 mg/kg); in addition, all the Feta samples showed histamine concentrations above the maximum permissible limits (100 mg/kg) suggested by Food and Drug Administration [140].

Few papers are present in the literature that concern amine content in soft cheeses obtained by mixture of milk breeds. Although scarce in number, it is possible to say that cheesemaking parameters deeply influence BAs accumulation, e.g., Ricotta Forte cheese.

## 5. Miscellaneous—Case Studies

This section discusses various studies focusing not only on one type of cheese, but on different types and the related BAs issues, focusing on the relationships occurring with microbiota, technological parameters, and surveys on multiple cheeses within the same or different categories. Table 2 summarizes the findings, indicating a high variability among samples attributed to factors such as milk composition, thermal treatment and microbial contamination.

Buňková et al. [146] observed higher BAs levels in ripened cheeses compared to fresh ones by processing more than hundred Czech Republic cheeses, with milk heat treatment showing varied effects depending on the type of milk. In fact, heat treatment did not influence the BAs content in ewe’s cheese, whereas differences were observed for goat milk products. Similar results were assessed by Fernández et al. [68], Novella-Rodríguez et al. [52] and Marino et al. [151], giving relevance on how heat treatment can be a suitable strategy to reduce the overall bacterial count and, particularly, of *Enterobacteriaceae* and enterococci. This evidence was further supported by Zdolec et al. [152], who processed and analyzed 69 cheese samples (8 mold-ripened cheeses, 18 semi-hard cheeses and 43 hard cheeses), observing a different BAs accumulation between the inner and outer parts of the cheese, and as it was mainly affected by the cheese type and microbial activity because enterococci and staphylococci significantly correlated with phenylethylamine, histamine and tyramine, whereas the *Enterobacteriaceae* count—indexing poor hygienic practices during cheesemaking—positively correlated with putrescine, phenylethylamine and histamine, while negatively correlated with spermine. Concerning the lower taxonomic levels, Dobranić et al. [153] and Celano et al. [154] reported that enterococci (*E. durans*, *E. faecium*, *E. faecalis*) are frequently found in raw milk, while Ladero et al. [155] enlightened the relationship occurring between tyramine and cheeses displaying *E. durans* as the dominant species.

However, the presence of BAs in cheese should not be strictly limited to these taxa alone. In fact, as said before, some authors have found commercial SLAB capable of producing BAs [43,151], while others provided opposite results [106,145,156,157]. This evidence takes its place on the BAs metabolism, which should be considered under a strain-dependent manner. In fact, some LAB, and molds and yeasts, were found to have a BAs reducing metabolism, being negatively correlated with histamine, putrescine and tyramine, in line with some cases reporting that hard and long-ripened cheeses showed lower BAs content than semi-hard cheeses. However, this conclusion differed from previously discussed findings where BAs production was highly related to ripening time and intense proteolytic activity exclusively. Also, Mayer & Fiechter [143] demonstrated different patterns in BAs accumulation. These authors highlighted the hazard of BAs accumulation occurring in acid-curd cheeses and hard cheeses, among the latter, mostly the “mountain cheese” (in which contaminant bacteria are often found) showed microorganisms with high decarboxylase activity, while extra-hard cheeses are completely safe due to the very low BAs levels found. Bonczar et al. [144] similarly concluded from their data that acid-curd short-ripened cheese (specifically Harzer cheese) had the highest amounts of amines, probably due to the high proteolysis process in comparison to other cheese-types such as Emmental, Cheddar, Camembert, Tvorog and fried cheese. Considering these debates, the reason was imputed to the cheese microbiota, which ascertains the competitiveness of SLAB against amine-producing NSLAB [158], as also concluded by Guarcello et al. [145] that found no linear correlation (but tendencies) between ripening time and BAs concentration in 20 different Italian cheeses, and this ambiguous result was discussed by the authors, giving relevance to the complex and multifaceted interplay of factors influencing BAs production. The accumulation of BAs varies across different cheese types and regions, with factors like microbial activity, different microbial niches in cheese wheel, ripening environment, and milk treatment influencing their concentration. Histamine content tends to increase with higher ripening temperatures, as shown by Madejska et al. [159] and StandaroVá et al. [160]. Rohani et al. [147] emphasize the need for hygiene control in cheese production to mitigate BAs-related risks to consumers. Ma et al. [139] assess BAs content in Egyptian cheeses, revealing correlations with microbial counts and identifying Mish cheese as particularly susceptible to spoilage. A serious problem was highlighted by Rohani et al. [147], who evaluated the BAs content in many samples of Koopeh, Lighvan and Red Salmas cheeses (typical West Azerbaijan cheeses). In fact, the results showed the total concentration ranged from 500 mg/kg (for Koopeh cheese) up to 1426 mg/kg (for Red Salmas cheese) of BAs. The authors concluded that it was necessary to control the hygienic conditions of both the raw material and the dairies, or at least the use of competitive adjunct culture is advisable.

Overall, these studies report conflicting conclusions, highlighting the complex interactions shaping BAs levels in cheese. In addition, the wide variability does not help in understanding their accumulation, as noted by Bonczar et al. [161], who found BAs content ranged from 4.4 to 2558 mg/kg in Pecorino cheese. Thus, given the complexity of the phenomenon, is not possible to provide absolute conclusions but broad tendencies, emphasizing the importance of a holistic understanding aimed at better managing these factors to ensure product safety and quality.

## 6. The Involvement of Cheese Starter Cultures in BAs Production—Case Studies

The role of starter cultures has been studied continuously since they were also discovered to be responsible for the BAs production. This section considers research papers published concerning the comparison between indigenous or commercial starters, as well as focused on findings demonstrating the ability of some adjunct culture to enhance the BAs production.

Knowing bacteria or other microorganisms responsible for amine production is essential, as it is possible to avoid the voluntary addition of them from the cheesemaking process. Ovalle-Marmolejo et al. [162] studied the potential producers of BAs among the LAB of ripened cheeses (from 3 to 8 months of ripening) and identified some strains of *L. mesenteroides* (from Manchego cheese) and *O. oeni* (from Cantal cheese) as possessing the gene codifying the decarboxylase useful to BAs production. Since *L. mesenteroides* is used as starter to improve the aroma of dairy products [163], it could represent a risk despite being found in mozzarella cheese and considered as possible probiotic bacteria [164]. By contrast, *O. oeni* is usually associated with wine products. The former was related to putrescine and tyramine formation, while the latter was related to putrescine and histamine production in their respective matrixes. del Valle et al. [149] isolated LAB from a ripened cheese made from raw goat milk inoculated or not with starter culture, aiming to evaluate a relationship between the producers of BAs and cheese BAs content. A higher LAB diversity was found in cheeses made without starter [49], with 60% of *Lactobacillus*, 32% of *Lactococcus* and 8% of *Leuconostoc*, whereas the cheese made with starter had ca. 30 different strains of *Lactobacillus*. By using the PCR technique, the authors searched for histidine decarboxylase-positive (hdc+) bacteria and demonstrated a higher presence of hdc+ strains in cheese without the starter (50% vs. 43.3%) to conclude about the importance of controlling the growth of autochthonous bacteria by mean of technological variables (pH, NaCl concentration, ripening time and temperature, cheesemaking and ripening environment). In general, the use of SLAB inhibits the growth of the indigenous microbiota since the latter is favored by the absence of competition.

However, as previously noted, the evidence also highlighted how SLAB possess the decarboxylase enzyme. To further investigate, Renes et al. [165] evaluated the effect of different SLAB and ripening time on BAs formation in a pasteurized ewe’s milk cheese. The results differed from the study of del Valle et al. [149]; these were in line with those obtained by Novella-Rodríguez et al. [166]. After 210 days of ripening, the cheese made with commercial SLAB showed the highest concentration of tryptamine, histamine, cadaverine and spermidine, whilst the others with indigenous SLAB reported reduced BAs formation. With differences affected by the core microbiota, batches of indigenous SLAB led to the highest amount of putrescine, spermine and phenylethylamine, and this has been related to the high *Enterobacteriaceae* count *Enterococcus* spp. found.

Some researchers focused their study only on one or few strains, for example, Flasarová et al. [167], who studied the BAs production by two strains of *Lc. lactis* subsp. *cremoris* (CCDM 824 and CCDM946) inoculated separately in two Dutch-type cheeses. Results indicated how these strains were tyramine and putrescine producers, as tyramine increased during the 90 days of ripening, with values above 370 and 500 mg/kg for CCDM824 and CCDM946, respectively, whereas putrescine followed the same trend reaching values above 800 mg/kg for both starters. To validate these data, the control sample showed the same amines at <5 mg/kg over time.

Egger et al. [168] studied the impact of added cultures of *L. helveticus* and milk heat treatment on raclette-type model cheese characteristics, obtaining 4 samples (1 raw milk cheese, 1 raw milk cheese + *L. helveticus*, 1 pasteurized cheese, 1 pasteurized cheese + *L. helveticus*). Raw milk cheeses showed the highest microbial diversity and BAs content (especially the one with added *L. helveticus* strains, which confirms the role of proteolysis on BAs formation) compared to pasteurized ones.

According to Torracca et al. [169], enterococci might be also involved in the BAs production. Rodríguez et al. [170] aimed to study *Tetragenococcus koreensis* and *T. halophilus* strains, as they are closely related to the *Enterococcus* genus taxonomically, isolated from two blue-veined cheeses (Picόn Bejes-tresviso and Cabrales), and searched for the presence of detrimental features, including resistance to antibiotics, genes responsible for pathogenicity or virulence factors, and decarboxylase enzymes. Fortunately, all of these factors were not present in the 20 strains of *Tetragenococcus* spp. studied, supporting their use as secondary cultures to enhance the typical bouquet of these cheese varieties.

Leuschner et al. [150] directly focused on the ability of enterococci to decarboxylate amino acids. Two of the tested strains (*E. faecalis*) were used as contaminants during Gouda cheese production and increased up to 10^9^ CFU g^−1^ during ripening. Starting from the second week of storage, tyramine (56 mg/kg) was found showing an uptrend up to ca. 470 mg/kg during the following 10 weeks. Also, putrescine was detected from the 8th to the 12th week of ripening at a much lower concentration (ca. 2 mg/kg), whereas no histamine was found. The control cheese showed no tyramine production but a similar content of putrescine. However, since experimental cheeses contaminated with *E. faecalis* showed a bitter off-flavor, the use of this strain as starter was not recommended. More recent work proved that biogenic amines production (i.e., putrescine, tyramine) is a species-level trait of *E. faecalis* [55].

Møller et al. [171] investigated various factors influencing histamine production during the ripening of cheddar cheese, including ripening temperatures (10 °C vs. 15 °C), salt content (normal or reduced) and the histamine-producing capability of NSLAB (*L. parabuchneri* KUH8). This particular bacterial species is typically undesirable in the dairy industry due to its association with histamine production in cheeses [172,173,174,175]. Additionally, all cheeses contained *L. helveticus* as SLAB, aimed at promoting proteolysis to facilitate the release of amino acids. The primary findings emphasized the significant role of salt in controlling the growth of indigenous bacteria, particularly in cheeses ripened at higher temperatures. Reduced-salt cheeses exhibited elevated histamine content, especially in cheeses ripened at higher temperatures (>3100 mg/kg), whereas normal salt content limited amine formation at lower temperatures. Therefore, both temperature and salt content were influential factors in histamine formation, consistent with the observations of Madejska et al. [159]. The impact of salt on the cheese environment may vary from cheese to cheese [176], leading to modifications in microbial growth, proteolysis, and consequently, amine formation. Regarding *L. parabuchneri* KUH8, it was inhibited by salt concentration during the early ripening phase, but no significant differences were observed between reduced and normal salt cheeses after 6 months. 

*L. parabuchneri* species cause concern not only due to the histamine production, but also for its ability to form biofilm on stainless steel, representing a cause of cheese contamination during or after the cheesemaking process [174,175]. According to Ascone et al. [173], the contamination source of such microorganisms occur often at farm level; thus, it is of pivotal importance to maintain high hygienic conditions throughout the entire production chain. Wechsler et al. [177] demonstrated that even a minimal inoculation level in raw matter could reflect in high levels of histamine formation in the corresponding cheese; however, it is inactivated under defined heat treatment (20–40 min at 56–57 °C), demonstrating curd heat treatment as a possible way to reduce the histamine level in cheese. In general, the authors stated that good hygiene conditions during milking are important to limit histamine formation in cheeses in which the curd is treated at lower temperature, and monitoring *L. parabuchneri* in raw milk could be considered a crucial step for raw milk cheeses producers, guaranteeing high quality and safe products.

Burdychova & Komprda [43] used the PCR method for screening for tyrosine and histidine decarboxylase genes in cheese-borne bacteria isolated from Dutch-type semi-hard cheese obtained using two different starter cultures. Of the 52 bacterial isolates selected (17 *Lactobacillus*, 19 *Enterococcus* and 16 *coliforms*), 17 were positive to PCR amplification, specifically 14 for tyrosine decarboxylase and 3 for the histamine decarboxylase coding gene. These bacteria were identified as *E. durans*, *E. faecalis*, *E. faecium* and *E. casseliflavus* as tyramine-producing strains, and *Lactobacillus curvatus*, *L. lactis* and *L. helveticus* as histamine-producing strains. This is noteworthy because the latter species is similar to that used as a SLAB; its voluntary addition is no longer recommended. By contrast, the other two *Lactobacillus* strains and all enterococci strains were not part of the starter cultures and originated from raw matter or contamination after milk heat treatment; the importance of strict observance of proper hygiene conditions during milk and cheese processing and handling was stressed.

## 7. The Involvement of Cheese-Isolated Eukaryotes in BAs Production—Case Studies

Bacteria are not the only living organisms able to produce BAs; yeasts can also contribute to the production of these molecules. Gardini et al. [59] studied the characteristics of forty-four yeast isolates involved in the manufacturing and ripening of Pecorino Crotonese cheese. Contrary to what was reported by Suzzi et al. [178], who stated that histamine production by yeasts is uncommon in dairy products, the in vitro results indicate that histamine and 2-phenylethylamine were mainly produced by the three strains of *D. hansenii*. Instead, the authors highlighted how the overall BAs production in Manteca cheese was supported by *Trichosporon asahii*, *C. rugosa* and *Rhodotorula mucilaginosa* metabolism, whereas Wyder et al. [179] mainly identified *Y. lipolytica*, *P. joadinii* and *D. hansenii* as the most BAs-producing yeast in wrapped Raclette cheese.

## 8. The Involvement of Microorganisms in Reducing BAs in Cheeses—Case Studies

The presence of BAs in foods represents a serious issue to solve during food production, maturation or storage. A possible strategy is to limit the release of amino acids; however, it is not suitable for cheeses as they contribute to the flavor and nutritional aspect of the product. Since BAs are resistant to cold or heating processes, another approach could be to limit their formation by using microorganisms without decarboxylase enzymes, but this entails huge efforts in bacteria characterization (mostly if they are natural (i.e., natural whey starters); moreover, hurdle technologies (hydrostatic pressures, irradiation, controlled-atmosphere packaging, additives) could be useful in controlling BAs formation; on the other hand, they can be expensive and impractical. Thus, with the aim of enhancing food security, many authors tried to evaluate microorganisms able to reduce the amine content in cheeses. The key characteristic to look for is the presence of amine-oxidizing enzymes that are responsible for amine degradation into H_2_O_2_, and the corresponding aldehyde and ammonia are useful to promote their own growth. Reports in the literature indicate many bacteria, molds and yeasts encoding for such enzymes [180]. Nowadays, to assess their best growing condition parameters, their ability to grow and reduce amines is under evaluation in vitro, whereas for other types of fermented food, their activity has been observed in wine [181], fish sauce [182] and sausages [183,184].

Concerning cheeses, Butor et al. [185] recently investigated the efficiency of a specific adjunct culture strain (*Bacillus subtilis* DEPE IB1) isolated from Gouda-type cheese as an amine-reducing bacteria in foods, and also searched for the environmental growing parameters enabling the highest efficiency in amine degradation. Other researchers have successfully tested the ability of *Bacillus polymyxa* D05-1, isolated from salted fish products, to reduce tyramine and histamine in Tallaga cheese experimentally contaminated with 40 mg/100 kg of amines, reporting 83.5 and 71.8% of histamine and tyramine reduction, respectively [186].

Also, different strains of *Brevibacterium linens* were tested for their histamine and tyramine degradation during Muenster cheese ripening [187]. Two out of three tested strains showed tyramine and histamine degradation over time, while the other strain only reduced tyramine content during the first 12 days of ripening, corresponding to its exponential growth phase. The same authors also found a BAs-reducing activity for *Geotrichum candidum* and coryneform bacteria isolated from cheese surfaces, and many others are reported for their ability to reduce histamine and tyramine in buffer solutions [187].

Herrero-Fresno et al. [188] isolated LAB from Zamorano, Cabrales and Emmental cheeses (long-ripened type) and found seventeen strains of *L. casei* showing histamine and tyramine reduction in broth culture. Then, the two best-performing strains were employed to evaluate their amine-reduction capability in a Cabrales-type cheese model. The outcomes showed good results in histamine and tyramine reduction, concluding that these two strains might be useful as adjunct starter cultures to mitigate the concentrations of BAs in cheeses. A similar study was conducted by Guarcello et al. [145], in which different strains of *Lactobacillus* were selected and successively employed as BAs reducing bacteria in Caciocavallo cheese, without modifying the typical sensory profile of the product. Adámek et al. [189] successfully obtained a reduced BAs concentration (putrescine, phenylethylamine, tyramine) in a Dutch-type cheese inoculated with added culture belonging to the *Lacticaseibacillus casei* and *Lactiplantibacillus plantarum* strains. The cheese added with *L. casei* CCDM198 showed a higher BAs reduction compared to the control cheese (32%), confirming this strain as the best among the tested microorganisms. Similar conclusions were drawn by Klementová et al. [190], who observed the ability of *Lacticaseibacillus casei* CCDM198 in reducing histamine, putrescine and cadaverine in vitro and in skimmed milk due to the multicopper oxidase enzyme activity. Such enzyme presence and activity in this species was first mentioned by these authors. As reported in a recent paper, Ramos et al. [191] obtained a reduction in total amines concentration up to 80% by using *L. acidophilus* UCLM-104 while preserving the sensory attributes of Manchego cheese.

Another strategy adopted by researchers is to use bacteriocins or bacteria-producing bacteriocins against BAs-producing bacteria [191,192]. Villarreal et al. [192,193] examined the antimicrobial ability of seven bacteriocins produced by LAB against 48 strains of histamine, tyramine and putrescine-producing bacteria. The results highlighted a good potential of bacteriocins in the control of BAs-producing LAB, with a perspective toward reducing the amine accumulation in cheeses.

## 9. Data Analysis

**Figure 2:**
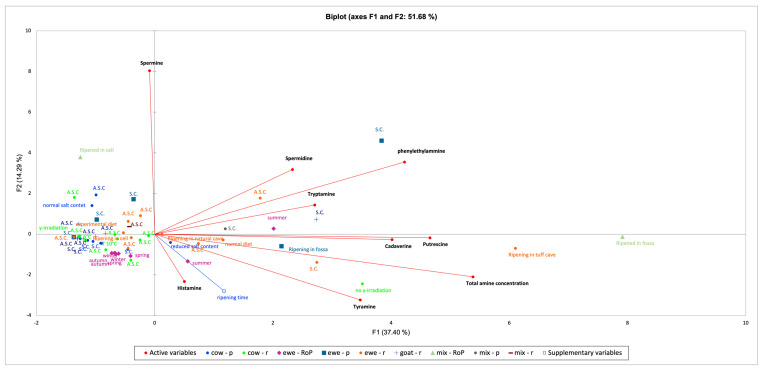
Multivariate analysis considering different factors influencing the BAs production in cheeses. Different symbols/colors indicate the milk origin species and milk thermal treatment, while labels near the symbols indicate the type of starter used, ripening environment or season, or treatment applied to the cheese (depends on the available information). Cow-p = cheese obtained from bovine pasteurized milk; cow-r = cheese obtained from bovine raw milk; ewe-RoP = cheese obtained from ewe raw or pasteurized milk; ewe-p = cheese obtained from ewe pasteurized milk; ewe-r = cheese obtained from ewe raw milk; goat-r = cheese obtained from goat raw milk; mix-RoP = cheese obtained from a mixture of bovine, ewe and goat raw or pasteurized milk; mix-p = cheese obtained from a mixture of bovine, ewe and goat pasteurized milk; mix-r = cheese obtained from a mixture of bovine, ewe and goat raw milk; A.S.C = autochthonous starter cultures; S.C = commercial starter cultures.

After reviewing these articles from the literature, the outcomes revealed many critical factors affecting the BAs content in cheeses. It is difficult to identify just one parameter as responsible of amines accumulation, as they arise due to the combination of different conditions, even though their role is sometimes questionable, as the literature reports show contrasting results. For this reason, an explorative multivariate statistical approach was used to better understand the presence of some pattern to help us unveil the amine accumulation in cheeses. By doing this, we have added one more factor that is always underestimated, the milk breed origin, which is responsible for many of the differences in raw matter and final products, as described in Section 2.4. The multivariate analysis applied was a principal component analysis (PCA) performed with Xlstat software (ver. 2023), with the following settings: (i) PCA type: correlation (Pearson); (ii) standardization: n-1, significance level (%): 5; (iii) type of biplot: correlation biplot; (iv) coefficient: automatic. The PCA was performed considering both qualitative and quantitative variables taken from the literature (Figure 2): (i) the qualitative variables were the presence of starter cultures (S.C.) or autochthonous cultures (A.S.C.), ripening time (as a supplementary variable), ripening environment (ripening in fossa, tuff cave, grotta, natural cave, cell), cheese treatment (γ-irradiation, no γ-irradiation), animal diet (normal diet, experimental diet), seasons (summer, winter, autumn, spring), milk breed (ewe, cow, goat or their combination) and milk thermal treatments (cheese exclusively obtained from raw or pasteurized milk, or those obtained indifferently from raw or pasteurized milk, “r”, “p” and “RoP”); (ii) the quantitative variables were the content of all eight biogenic amines reported in the literature (when available).

The biplot explained 51.68% of the total variance, in which the first component (PC1) accounted for 37.4% and the second component (PC2) for 14.29%. The active variables, representing the BAs, extend towards the positive side of PC1 (except for spermine); thus, all the samples placed in these quadrants have a higher content of such molecules, while the others plotting on the back had lower BAs amounts. The biplot showed interesting results. It is possible to observe the negative influence of reducing salt in cheese, mostly if it is made by using A.S.C., which refers to natural whey starters or spontaneous fermentation. The reduced-salt cheese sample plots on the positive side of PC1, meaning that it accumulated higher BAs than its corresponding sample, “normal salt content” (IV quadrant, upper left side). The season greatly influenced the amine formation, as summer samples plot in the first and second quadrants, while all the other samples (winter, autumn, spring) plot on the back of the active variables. The γ-irradiation treatment shows good results, as its control (no γ-irradiation) plots on the opposite side (II quadrant) with respect to the treated one. The same results were obtained for the “experimental diet” (added with 10% of grape pomace) compared to the “normal diet”.

In our opinion, one of the most important factors in amine formation is the ripening environment, as all the cheeses ripened in uncontrolled environments plot on the positive side of PC1, whereas the sample ripened in controlled cell plots on the negative side. Moreover, as predicted, it is important to state that most of these samples were obtained from ewe milk, which could be more contaminated with BAs-producing bacteria. The samples ripened in the fossa and tuff cave, obtained from the ewe and breed mixture milk, showed the highest BAs concentration. An unclear separation is observed when considering the use of starter cultures or autochthonous starter cultures. This is expected, since they could vary greatly in number, composition and characteristics; thus, it is possible to find cheeses obtained with commercial starter cultures in the I and II quadrants (rich in BAs) and those obtained with autochthonous starter cultures in the III and IV quadrants, and vice versa. An important, pivotal factor when considering the commercial starter cultures is to assess if the specific strain has the decarboxylase enzymes, in which case they can possibly contribute to the amine accumulation in cheese. The other two parameters considered are the milk heat treatment and milk breed, which influence the BAs content but to a lesser extent than the other factors, as samples are well mixed, and their distribution reflects more of the effect from the other parameters. Finally, the ripening time appears as a supplementary variable (as its effect is predictable), and extends towards the highest amine concentration, meaning that it has an impact on their formation.

## 10. Final Considerations and Future Perspectives

The holistic approach used herein can highlight some aspects of amine accumulation in cheese that can give useful information on the phenomena that suggest strategies or solutions capable in mitigating contamination. All the parameters considered are directly or indirectly related to microbial activity. Concerning the level of salt in the product, which clearly reduces the amines content, its consequence is the influence on the evolution of microbial populations [132]. Of course, using large amounts of salt to solve the problem of amine accumulation is not feasible, due to known issues on human health such as cardiovascular diseases. The use of hurdle technologies could mitigate BAs formation or even directly reduce their content in the product [124]; nevertheless, the feasibility of the applied technology and the costs must be carefully considered. Also, using grape pomace as an animal food supplement should be expanded because it provides for (i) use of byproducts in a circular economy and (ii) amine profile reduction [117]. Another pivotal factor influencing the amine formation in cheese is the ripening environment, since uncontrolled conditions favor the contamination and the growth of specific microorganisms that can easily produce BAs [7,8,109,110,111,112]. Still considering the microbial population of the cheese, the influence of dairy species and the quality of milk could favor or limit the amine production [107,122]. On the other hand, the absolute certainty of milk heat treatment as an amine reduction strategy is questioned, as results reported in the literature are contradictory [52,68,146]. Finally, it is very important to select the proper microorganism to be used as the starter, as some producers of BAs can also be found among those defined as GRAS.

In conclusion, BAs formation is a complex phenomenon, influenced by many factors to varying degrees, but all of them must be considered to control amine formation and accumulation in cheese. A suitable strategy to limit the excessive ingestion of these molecules by consumers should involve both controlling all the cheesemaking parameters and consuming high-quality products (i.e., with low contamination from exogenous microorganisms, high-quality raw matter, good hygiene practices, controlled cheesemaking process). However, each cheese has its own peculiar technological history (type of starter, processing technology, ripening environment, etc.), and proposing a generic solution for amine mitigation cannot fit all cheese types. For this reason, as a future perspective, it is important to carefully evaluate each product to find a suitable solution, modeling the cheesemaking parameters that most closely fit the product without influencing its sensory characteristics. In parallel, the research field of bacteria-consuming amines could be a good starting point for testing them in different cheeses to evaluate their effect on amines and their impact on the sensory properties of the final product.

## Figures and Tables

**Table 1:** Amine chemical characteristics and health properties/issues. Erdag et al. [22], Benkerroum [33] and Alvarez & Moreno-Arribas [34].

Name	Precursor	Structure	Body Function *	Health Issues
Histamine	Histidine	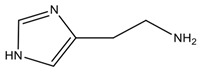 Aromatic	Balancing the body temperature and regulating the stomach volume, stomach pH, cerebral activities, neurotransmitter and inflammation mediators; it also initiates allergic reactions	Responsible for “scombrid poisoning”, hypotension, headache, abdominal cramps, diarrhea and vomiting
Tyramine	Tyrosine	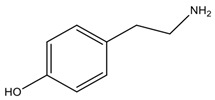 Aromatic	Vasoconstriction *, increases blood pressure, active noradrenalin secretion, inflammation mediators	Responsible for the “cheese reaction”, hypertensive crises and headache; it can inhibit some oxidase enzymes
Tryptamine	Tryptophan	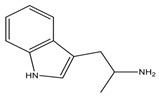 Aromatic	Vasoconstriction *, increases blood pressure, neurotransmitter and neuromodulator	Increases blood pressure, hypertension
Phenylethylamine	Phenylalanine	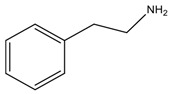 Aromatic	Neurotransmitter	Hypertensive crises and headache
Putrescine	Ornithine, glutamine, arginine, agmatine	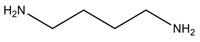 Diamine	Found in high concentration in brain; a deficiency led to depression syndrome *; regulation of gene expression, maturation of intestine, cell growth and differentiation	Promotes the formation of nitrosamines, cadaverine and polyamines; inhibits oxidase enzymes, enhancing the toxicity of histamine and tyramine; causes hypotension, bradycardia and lockjaw
Cadaverine	Lysine	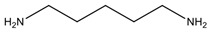 Diamine	Regulation of gene expression, maturation of intestine, cell growth and differentiation	Promotes the formation of nitrosamines; inhibits oxidase enzymes, enhancing the toxicity of histamine and tyramine
Spermidine	Putrescine + propylamine residue from S-adenosyl-methionine	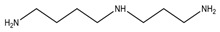 Polyamine	Growth regulation; regulation of membrane-linked enzymes *	Associated with food allergies; could promote colon cancer.
Spermine	spermidine + propylamine residue from S-adenosyl-methionine	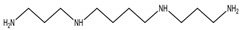 Polyamine	Growth regulation; regulation of membrane-linked enzymes	Associated with food allergies; could promote colon cancer

* Some medical aspects concern animal studies.

**Table 2:** Biogenic amines content reported in published research, expressed as mg/kg.

Cheese Name	Species	Milk Heat Treatment	Variable Considered	Ripening Time (Days)	His	Tyr	Put	Cad	Try	Sme	Spd	Phe	Reference
Montasio	Cow	Raw		60	1.9 (1.7)	3.33 (3.6)	19.19 (36.9)	3.22 (6.1)	0.19 (0.3)	0 (0)	0 (0)	0.46 (0.5)	[99]
				90	3.7 (1.6)	5.19 (2.8)	1.15 (1.2)	0.49 (0.4)	0.14 (0.1)	0 (0)	0 (0)	0.42 (0.6)	
				120	6.71 (5)	6.09 (3.5)	0.68 (0.6)	0.22 (0.2)	0.12 (0.1)	0 (0)	0 (0)	0.47 (0.4)	
				150	21.66 (11)	24.89 (10.1)	36.36 (33.1)	1.3 (1.1)	0.4 (0.2)	0 (0)	0 (0)	1.42 (0.4)	
Toma Piemontese PDO	Cow	Raw	not commercial	4	12.00	10.00	0.00	0.00	0.00	0.00	0.00	0.00	[65]
				19	14.00	26.00	0.00	0.00	0.00	0.00	0.00	0.00	
				29	15.00	34.00	0.00	0.00	0.00	0.00	0.00	0.00	
				39	22.00	60.00	0.00	0.00	0.00	0.00	0.00	0.00	
				49	37.00	108.00	0.00	0.00	0.00	0.00	0.00	0.00	
			commercial		0.00	39.00	0.00	0.00	0.00	0.00	0.00	0.00	
					0.00	178.00	0.00	0.00	0.00	0.00	0.00	0.00	
					40.00	95.00	0.00	0.00	0.00	0.00	0.00	0.00	
					67.00	58.00	0.00	0.00	0.00	0.00	0.00	0.00	
					29.00	79.00	0.00	0.00	0.00	0.00	0.00	0.00	
					0.00	0.00	0.00	0.00	0.00	0.00	0.00	0.00	
					0.00	146.00	0.00	0.00	0.00	0.00	0.00	0.00	
					54.00	165.00	0.00	0.00	0.00	0.00	0.00	0.00	
					27.00	31.00	0.00	0.00	0.00	0.00	0.00	0.00	
Cheddar	Cow				33.40	52.90	19.40	13.90	6.90	0.00	0.00	0.00	[141]
Ras					82.80	43.20	74.40	36.80	28.30	24.00	0.00	0.00	
Chihuahua cheese	Cow			0	0 (0)	33 (47.5)	0 (0)	0 (0)	0 (0)	0 (0)	0 (0)	0 (0)	[101]
				60	7.5 (18.4)	110.83 (66.2)	0 (0)	0 (0)	0 (0)	0 (0)	0 (0)	0 (0)	
				120	50.33 (74.7)	173.17 (36.6)	0 (0)	0 (0)	0 (0)	0 (0)	0 (0)	0 (0)	
Pecorino Abruzzese	Ewe	Raw	autochthonous starter culture	0	0.00	0.00	0.00	0.00	0.00	0.00	0.00	0.00	[106]
				14	20.00	100.00	55.00	10.00	5.00	5.00	6.00	50.00	
				30	100.00	80.00	10.00	10.00	20.00	5.00	5.00	20.00	
				60	280.00	190.00	50.00	60.00	25.00	5.00	50.00	30.00	
		Past.	commercial starter cultures (*Streptococcus thermophilus*, *Lactobacillus casei* and *Lb. delbruekii* subsp. *bulgaricus*)	0	0.00	0.00	0.00	0.00	0.00	0.00	0.00	0.00	
				14	30.00	10.00	10.00	2.00	10.00	5.00	4.00	5.00	
				30	120.00	10.00	10.00	10.00	20.00	10.00	15.00	10.00	
				60	110.00	280.00	190.00	100.00	30.00	20.00	20.00	300.00	
Formaggio di Fossa	Mix		ripened in Fossa	100	24.00	461.00	579.60	1302.86	0.00	0.00	16.49	173.00	[111]
			ripened in cell	100	0.00	24.92	18.57	0.00	0.00	27.58	0.00	0.00	
Ewe cheese	Ewe	Raw	Commercial starter (*Lactococcus lactis*, *Lactococcus cremoris*)	1	13.80	27.40	30.10	41.00	22.10	0.00	20.50	13.00	[105]
				15	25.60	40.30	66.40	70.90	31.00	0.00	29.90	22.20	
				30	11.50	35.10	63.60	70.70	12.30	0.00	15.30	11.20	
				90	0.00	88.60	98.40	78.10	0.00	0.00	0.00	0.00	
				120	0.00	101.00	70.40	65.70	0.00	0.00	0.00	0.00	
				180	0.00	238.00	103.30	77.10	0.00	0.00	0.00	0.00	
Herby cheese	Ewe			360	197.9 (155.32)	360.39 (253.66)	192.51 (209.23)	288.46 (435.40)	103.3 (37.09)	0 (0)	0 (0)	33.58 (31.79)	[108]
Pecorino di farindola	Ewe			90	5.18 (7.27)	417.73 (362.49)	119.82 (145.16)	100.46 (78.53)	0 (0)	0 (0)	78.94 (37.93)	31.74 (39.01)	[112]
Terrincho cheese	Ewe		market cheese	30	4.04 (5.56)	79.86 (117.24)	218.48 (142.18)	135.6 (78.61)	67.68 (59.11)	0 (0)	0 (0)	65.84 (95.88)	[115]
Pecorino	Ewe	Past.	natural cave ripening	150	0.00	420.00	22.00	2.00	11.00	2.00	0.00	56.00	[109]
		Raw	ripening room	60	0.00	147.00	54.00	37.00	12.00	0.00	0.00	24.00	
		Past.	ripened in fossa	180	10.00	1040.00	39.00	54.00	12.00	1.00	0.00	127.00	
		Raw	tuff cave	150	23.00	1132.00	512.00	262.00	88.00	0.00	0.00	144.00	
Canestrato di Castel del Monte	Ewe	Past.	no starter culture	240	10.30	0.00	0.00	0.00	0.00	0.00	0.00	0.00	[53]
Pecorino di fossa	Mix	Raw		90	743.30	1771.30	986.00	2127.00	0.00	0.00	0.00	232.40	
Pecorino sotto fieno	Ewe	Past.		180	200.00	58.90	8.30	36.90	0.00	0.00	0.00	0.00	
Pecorino di grotta	Mix	Past.		120	235.40	312.10	8.90	0.00	0.00	0.00	0.00	26.00	
Pecorino abruzzese sott’olio		Raw		240	13.10	394.50	36.30	57.30	0.00	0.00	0.00	62.10	
Pecorino di Atri or Abruzzese	Ewe	Raw		150	0.00	10.60	0.00	0.00	0.00	0.00	0.00	0.00	
Pecorino d’Abruzzo				300	130.70	140.90	128.10	33.60	0.00	0.00	0.00	12.70	
Pecorino d’Abruzzo				90	266.70	0.00	0.00	0.00	0.00	0.00	0.00	0.00	
Pecorino d’Abruzzo					186.00	702.40	377.70	172.40	0.00	0.00	0.00	44.40	
Pecorino d’Abruzzo					14.50	224.40	369.00	116.40	0.00	0.00	0.00	0.00	
Pecorino d’Abruzzo					761.00	7.70	55.00	0.00	0.00	0.00	0.00	0.00	
Pecorino di Farindola					11.20	397.70	127.00	110.60	0.00	0.00	0.00	0.00	
Zamorano PDO	Ewe	Raw		1	10.00	0.00	15.00	35.00	0.00	0.00	0.00	8.00	[107]
				60	10.00	30.00	40.00	36.00	10.00	3.00	30.00	14.00	
				120	18.00	41.00	48.00	40.00	15.00	10.00	38.00	51.00	
				180	20.00	48.00	51.00	41.00	20.00	21.00	50.00	55.00	
				240	40.00	52.00	100.00	44.00	27.00	20.00	53.00	130.00	
				300	42.00	76.00	131.00	45.00	44.00	21.00	100.00	149.00	
		Past.		1	10.00	1.00	14.00	35.00	3.00	1.00	0.00	2.00	
				60	11.00	19.00	13.00	35.00	4.00	21.00	1.00	4.00	
				120	11.00	19.00	14.00	36.00	11.00	21.00	2.00	12.00	
				180	12.00	31.00	14.00	38.00	11.00	22.00	3.00	13.00	
				240	18.00	39.00	18.00	37.00	18.00	28.00	3.00	21.00	
				300	25.00	42.00	19.00	37.00	22.00	31.00	4.00	25.00	
Ewe cheese	Ewe	Past.	summer	100	134.77	185.19	35.26	191.59	124.17	0.00	0.00	69.68	[113]
			summer	180	233.42	501.65	6.61	149.44	0.00	0.00	0.00	19.63	
			autumn	100	234.14	152.61	0.00	6.39	0.00	0.00	0.00	0.00	
			autumn	180	211.19	210.90	0.00	1.28	0.00	0.00	0.00	0.00	
			winter	100	237.59	183.48	0.00	6.39	0.00	0.00	0.00	0.00	
			winter	180	204.24	234.92	0.00	7.66	0.00	0.00	0.00	0.00	
			spring	100	202.85	209.20	0.00	1.28	0.00	0.00	0.00	0.00	
			spring	180	202.85	338.24	0.00	1.28	2.00	0.00	0.00	0.00	
Pecorino	Ewe	Raw	normal diet	2	0.00	0.00	0.00	0.00	0.00	0.00	0.00	0.00	[117]
			10% grape pomace-enriched diet		0.00	0.00	0.00	0.00	0.00	0.00	0.00	0.00	
			normal diet	60	0.00	32.53	0.00	0.00	0.00	0.00	0.00	38.11	
			10% grape pomace-enriched diet		0.00	42.72	7.63	18.33	0.00	0.00	0.00	13.35	
			normal diet	90	0.00	40.18	0.00	0.00	0.00	0.00	0.00	46.28	
			10% grape pomace-enriched diet		0.00	34.55	5.34	10.67	0.00	0.00	0.00	15.04	
			normal diet	120	0.00	101.23	0.00	15.74	0.00	0.00	0.00	85.37	
			10% grape pomace-enriched diet		0.00	103.03	14.11	20.00	0.00	0.00	0.00	63.51	
Semicotto Caprino	Goat	Raw		15	0.00	50.00	100.00	20.00	30.00	0.00	0.00	10.00	[118]
				30	50.00	120.00	220.00	40.00	90.00	0.00	40.00	40.00	
				60	130.00	230.00	940.00	100.00	380.00	20.00	70.00	140.00	
Goat cheese	Goat	Raw		1	3.05	4.49	10.00	53.47	1.29	0.00	0.00	0.80	[52]
				14	3.45	29.25	33.97	72.04	3.38	0.00	0.00	2.62	
				30	5.37	93.47	42.86	87.15	7.00	0.00	0.00	6.26	
				60	27.99	245.32	74.15	177.81	9.05	0.00	0.00	19.64	
				90	43.06	324.67	86.40	196.47	12.15	0.00	0.00	27.34	
		Past.		1	0.00	0.00	0.79	1.29	0.00	0.00	0.00	0.70	
				14	1.19	0.96	3.86	1.93	2.21	0.00	0.00	1.48	
				30	2.16	2.54	7.83	5.77	5.42	0.00	0.00	4.46	
				60	3.50	7.22	8.17	15.52	6.48	0.00	0.00	7.97	
				90	6.34	10.90	14.61	32.73	7.56	0.00	0.00	8.89	
					41.90	43.60	5.60	74.80	3.00	0.00	0.00	6.20	[119]
					26.40	37.00	10.60	2.60	3.80	0.00	0.00	22.50	
					10.20	50.70	2.20	0.50	11.90	0.00	0.00	3.80	
					21.00	14.10	0.80	1.10	0.00	0.00	0.00	1.90	
					40.90	45.20	2.80	0.70	1.20	0.00	0.00	1.30	
					60.50	22.40	1.60	7.20	0.00	0.00	0.00	0.80	
					18.90	4.20	0.90	1.50	0.00	0.00	0.00	1.00	
					28.50	45.30	21.70	1.30	0.00	0.00	0.00	2.20	
Ricotta Forte	Mix		control (no sorbic acid)		1256.96	2797.78	1469.10	3759.50	0.00	19.34	12.00	491.33	[137]
	Ewe		with preservative (sorbic acid)		201.44	387.77	431.68	1044.61	0.00	21.12	0.00	0.00	
	Ewe		control (no sorbic acid)		1273.51	2142.08	1468.51	4641.05	0.00	29.79	17.27	721.80	
Blue cheese	Cow	Raw	no γ-irradiation	0	15.70	980.00	14.80	7.20	0.00	0.00	5.40	0.00	[124]
			γ-irradiation 2kGy	0	16.50	985.00	15.70	9.20	0.00	0.00	10.30	0.00	
			γ-irradiation 4kGy	0	16.80	969.00	17.00	6.70	0.00	0.00	10.80	0.00	
			γ-irradiation 6kGy	0	0.00	950.00	7.60	3.30	0.00	0.00	15.40	0.00	
			no γ-irradiation	30	22.80	1021.00	18.90	16.10	0.00	0.00	6.10	0.00	
			γ-irradiation 2kGy	30	18.50	877.00	14.50	7.50	0.00	0.00	4.00	0.00	
			γ-irradiation 4kGy	30	16.90	588.00	13.10	4.30	0.00	0.00	6.00	0.00	
			γ-irradiation 6kGy	30	0.00	563.00	7.30	3.80	0.00	0.00	7.00	0.00	
			no γ-irradiation	60	23.20	1775.00	21.70	9.20	0.00	0.00	8.40	0.00	
			γ-irradiation 2kGy	60	22.50	834.00	15.10	8.30	0.00	0.00	6.00	0.00	
			γ-irradiation 4kGy	60	9.10	646.00	12.20	2.30	0.00	0.00	12.80	0.00	
			γ-irradiation 6kGy	60	0.00	439.00	9.50	2.40	0.00	0.00	10.10	0.00	
			no γ-irradiation	90	35.70	2219.00	26.50	12.30	0.00	0.00	15.90	0.00	
			γ-irradiation 2kGy	90	29.00	978.00	22.60	9.30	0.00	0.00	14.80	0.00	
			γ-irradiation 4kGy	90	1.80	626.00	19.20	3.20	0.00	0.00	15.00	0.00	
			γ-irradiation 6kGy	90	0.00	403.00	9.10	3.90	0.00	0.00	15.40	0.00	
Niva	Cow		market	30	17.33 (4.91)	77.9 (66.88)	24.55 (7.06)	188 (185.88)	3.45 (2.07)	2.23 (0.41)	3.5 (1.46)	0 (0)	[127]
Gamalost	Cow			0	0.00	0.00	0.00	0.00	0.00	0.00	0.00	0.00	[130]
				10	0.00	0.00	11.87	0.00	0.00	0.00	0.00	0.00	
				20	0.00	0.00	25.17	0.00	0.00	0.00	0.00	0.00	
				30	0.00	0.00	24.57	0.00	0.00	0.00	0.00	0.00	
				60	0.00	0.00	16.15	0.00	0.00	0.00	0.00	0.00	
Norvegia cheese				90	1.43	5.56	0.00	0.00	0.00	1.09	0.00	0.00	
Surface-ripened cheeses	Cow		reduced salt content	27	0.00	0.00	54.10	460.30	0.00	0.00	0.00	0.00	[132]
			normal salt content	27	0.00	0.00	11.40	153.80	0.00	0.00	0.00	0.00	
Olomouc curd cheese	Cow	Past.	storage temperature 5 °C		150.00	320.00	290.00	160.00	20.00	10.00	20.00	10.00	[133]
			storage temperature 20 °C		160.00	1350.00	800.00	1430.00	160.00	10.00	10.00	160.00	
			shape disk		200.00	840.00	360.00	550.00	100.00	10.00	20.00	150.00	
			shape bar		75.00	415.00	370.00	490.00	180.00	10.00	10.00	100.00	
				8	0.00	160.00	160.00	80.00	0.00	0.00	0.00	0.00	
				10	0.00	390.00	160.00	120.00	0.00	0.00	0.00	0.00	
				42	0.00	790.00	410.00	640.00	0.00	0.00	0.00	0.00	
				66	0.00	880.00	610.00	830.00	0.00	0.00	0.00	0.00	
			winter		60.00	620.00	280.00	710.00	200.00	5.00	10.00	110.00	
			spring		240.00	740.00	430.00	300.00	90.00	10.00	20.00	99.00	
			summer		90.00	480.00	390.00	550.00	110.00	3.00	15.00	180.00	
Civil cheese	Cow	Raw		0	0.00	0.95	0.34	2.31	0.52	0.26	0.26	0.66	[128]
			ripening temperature 5 °C	30	0.00	0.84	0.13	1.40	0.04	0.07	0.27	0.75	
			ripening temperature 10 °C	30	0.00	5.34	1.80	2.54	0.07	0.10	0.37	0.54	
			ripening temperature 5 °C	60	0.00	2.22	0.40	2.25	0.06	0.26	0.21	0.76	
			ripening temperature 10 °C	60	0.00	18.55	32.20	106.42	0.60	0.12	0.31	2.93	
			ripening temperature 5 °C	90	0.00	4.12	1.85	2.87	0.07	0.04	0.36	6.24	
			ripening temperature 10 °C	90	0.00	19.93	47.79	151.41	0.89	0.10	0.43	1.06	
Camembert	Cow	Past.	mold strain type 1	14	12.34	0.00	15.60	12.61	0.00	0.00	18.13	0.00	[135]
			mold strain type 2		11.57	0.00	15.83	11.91	0.00	0.00	17.32	0.00	
			mold strain type 3		11.23	0.00	14.55	12.21	0.00	0.00	20.43	0.00	
			coagulant type 1		10.59	0.00	15.40	13.55	0.00	0.00	20.45	0.00	
			coagulant type 2		10.75	19.38	15.46	13.27	0.00	0.00	20.02	0.00	
			coagulant type 3		11.23	20.38	16.12	14.71	0.00	0.00	21.69	0.00	
Burrata	Cow				0.00	0.00	0.00	0.00	0.00	9.27	0.00	0.00	[142]
Mascarpone					0.00	0.00	0.00	0.00	0.00	7.24	0.00	0.00	
Cream					0.00	25.66	9.57	41.07	0.00	8.85	0.00	24.47	
Ricotta					0.00	0.00	0.00	0.00	0.00	11.16	0.00	12.12	
Pecorino Romano	Ewe				1468.46	125.00	99.43	108.20	0.00	88.65	0.00	0.00	
Parmigiano Reggiano	Cow				0.00	31.81	0.00	5.07	0.00	90.11	0.00	0.00	
Grana Padano					68.51	48.36	49.84	4.13	0.00	66.95	0.00	0.00	
Asiago					7.92	110.93	23.06	18.27	14.00	15.53	0.00	15.02	
Mimolette					0.00	96.32	0.00	65.19	17.88	104.01	8.71	16.75	
Gouda					24.15	12.70	0.00	152.92	0.00	23.11	5.25	9.63	
Cheddar					176.87	63.03	7.86	18.88	14.67	56.34	0.00	15.94	
Gruyere					22.25	52.47	0.00	17.04	33.27	125.53	0.00	0.00	
Emmental					0.00	81.98	22.64	13.26	0.00	50.48	4.27	12.63	
Smoked					0.00	30.99	8.22	13.06	0.00	42.74	0.00	0.00	
Comte					8.69	149.13	0.00	10.27	0.00	101.29	0.00	10.03	
Edam					12.79	209.20	20.61	41.83	0.00	73.05	10.80	26.56	
Appenzeller					225.69	101.69	13.77	58.08	0.00	153.23	0.00	0.00	
Camembert					7.23	11.53	6.92	0.00	0.00	15.20	26.65	22.25	
Brie					8.67	12.81	7.43	0.00	0.00	17.12	15.87	9.90	
Feta	Goat				14.27	310.00	100.00	3.00	9.82	12.80	17.35	5.22	
Chevre					0.00	0.00	0.00	0.00	0.00	11.28	0.00	0.00	
Caprice des dieux	Cow				0.00	19.40	8.01	0.00	0.00	59.97	14.39	0.00	
Bleu d’Auvergne					4.30	22.93	13.57	0.00	0.00	105.99	14.70	0.00	
Fauquet Maroilles					0.00	90.66	15.35	14.18	0.00	132.11	13.67	8.90	
Extra-hard cheeses					51	4.6	1.7	2.7	3.3	0.0	0.0	0.0	[143]
Hard cheeses					204.9	97.5	18.8	16.5	9.3	0.0	0.0	0.0	
Semi-hard cheeses					38.3	151.7	36.5	47.3	49.3	0.0	0.0	0.0	
Blue-veined cheeses					36.6	13.9	15.9	9.5	3.5	0.0	0.0	0.0	
Mold-ripened soft cheeses					4.0	3.1	26.8	67.2	12.7	0.0	0.0	0.0	
Smear-ripened cheeses					17.9	47.1	23.0	8.1	5.4	0.0	0.0	0.0	
Acid-curd cheeses					28.8	156.4	118.3	86.9	14.9	0.0	0.0	0.0	
Cheddar	Cow		rennet curd		0.00	0.00	0.00	0.00	0.00	20.20	25.10	0.00	[144]
Emmentaler			rennet curd		5.80	5.78	2.67	1.60	0.90	2.79	2.29	2.08	
Camembert			rennet curd		2.45	67.58	67.02	4.33	1.21	1.89	1.67	8.76	
Tvorog			acid-curd		1.08	6.23	6.47	2.43	0.92	1.02	2.82	0.87	
Harzer			acid-curd		1.08	6.23	6.47	2.43	0.92	1.02	2.82	0.87	
Fried			acid-curd		24.08	275.50	281.33	377.50	48.91	5.70	7.74	5.30	
Vastedda della Valle del Belice PDO	Ewe	Raw	no starter culture	2	5.70	5.90	1.10	1.00	1.40	1.10	0.30	0.50	[145]
Caprino di Castel Fiorentin	Goat		natural whey starter	30	0.00	25.00	9.00	0.00	0.00	4.70	0.00	0.00	
Caciocavallo Podolico Dauno	Cow		natural whey starter	30	0.00	7.00	0.00	1.40	0.00	13.60	0.00	0.00	
Fior di Capra	Goat		no starter culture	90	18.00	29.30	15.40	0.30	0.80	0.00	0.00	0.00	
Caprino Girgentano			no starter culture	90	89.00	1.70	6.20	0.90	0.40	0.00	0.00	4.00	
Caprino di Biccari		Thermised	starter cultures	210	44.00	227.00	298.00	27.00	73.00	0.00	5.00	145.00	
Caciocavallo Palermitano	Cow	Raw	no starter culture	120	149.00	4.00	12.00	0.00	4.00	0.00	13.00	0.00	
Ragusano PDO			no starter culture	120	54.80	23.90	7.60	2.90	0.00	0.00	0.00	4.50	
Caciocavallo Silano PDO			natural whey starter	150	435.00	33.00	37.00	17.00	6.00	0.00	0.00	0.00	
Cacioricotta		Past.	no starter culture	30	19.00	13.00	14.00	18.00	0.00	10.00	5.00	0.00	
Fiore Sicano		Raw	no starter culture	60	155.00	32.00	16.00	32.00	53.00	0.00	0.00	2.60	
Cacio	Mix	Past.	starter cultures	60	17.00	305.00	53.00	111.00	18.00	0.00	0.00	143.00	
Vaccino	Cow		no starter culture	90	14.90	4.30	62.00	28.00	0.00	15.00	0.00	0.00	
Provola dei Nebrodi		Thermised	no starter culture	30	1.00	1.00	0.75	0.40	0.10	0.00	0.10	0.10	
Pecorino Foggiano	Ewe		starter cultures	120	253.00	303.00	594.00	129.00	0.00	0.00	0.00	25.00	
Canestrato Pugliese PDO		Raw	no starter culture	90	3.90	273.00	208.00	199.00	0.00	0.00	4.00	46.00	
Maiorchino	Mix	Thermised	no starter culture	180	2.00	61.00	5.00	1.30	1.90	0.00	3.50	82.00	
Pecorino Siciliano PDO	Ewe	Raw	no starter culture	120	219.00	86.00	60.00	5.40	2.80	0.00	2.00	9.00	
Piacentinu Ennese			no starter culture	270	243.00	134.00	204.00	9.50	6.00	0.00	10.00	28.50	
Tuma Persa	Cow	Thermised	no starter culture	270	250.00	14.00	23.00	0.00	2.30	0.00	0.00	4.00	
Bryndza	Ewe	Raw			24.2	107.4	60.9	42.6	0.0	9.7	0.00	0.0	[146]
Smoked cheese					0.0	38.3	99.9	80.7	0.0	0.0	0.0	0.0	
Fresh cheese					0.0	0.0	20.7	19.6	0.0	0.0	0.0	0.0	
Unripened (fresh) cheese		Past.			0.0	11.1	118.2	35.8	0.0	0.0	0.0	0.0	
Pasta filata-type cheese					0.0	0.0	0.0	0.0	0.0	13.2	0.0	0.0	
Brined cheese					0.0	174.6	229.5	125.6	0.0	14.0	0.0	0.0	
Flavored cheese					0.0	114.7	108.8	0.0	0.0	0.0	0.0	0.0	
Ripened cheese	Goat	Raw			0.0	207.1	0.0	149.0	0.0	0.0	0.0	0.0	
Unripened (fresh) cheese (unflavored)		Past.			0.0	11.3	0.0	0.0	0.0	0.0	0.0	0.0	
Unripened (fresh) cheese (flavored)					0.0	10.7	0.0	0.0	0.0	0.0	0.0	0.0	
Pasta filata type cheese					0.0	8.5	41.1	40.3	0.0	0.0	0.0	0.0	
Unripened (fresh) cheese (unflavored)	Cow	Past.			0.0	15.2	111.4	8.9	0.0	17.9	0.0	0.0	
Unripened (fresh) cheese (flavored)					0.0	22.7	0.0	22.4	0.0	15.5	0.0	0.0	
Ripened cheese					0.0	101.4	70.1	72.6	0.0	97.9	0.0	0.0	
Pasta filata type cheese (unflavored)					19.3	25.8	37.3	0.0	0.0	12.7	0.0	0.0	
Pasta filata type cheese (flavored)					0.0	0.0	14.7	0.0	0.0	12.0	0.0	0.0	
Pasta filata type cheese (unflavored, smoked)					18.8	0.0	0.0	10.7	0.0	9.8	0.0	0.0	
Koopeh	Cow				70.80	8.48	156.09	282.34	0.00	0.00	0.00	0.00	[147]
Lighvan					37.59	351.12	277.53	342.74	0.00	0.00	0.00	0.00	
Red Salmas					105.21	182.62	438.03	701.05	0.00	0.00	0.00	0.00	
White-veined cheese	Cow				0 (0)	15.69 (8.53)	25.22 (35.14)	34.57 (103.7)	0 (0)	37.23 (17.43)	1.83 (2.76)	0 (0)	[148]
Blue-veined cheese					0 (0)	101.02 (65.09)	12.62 (6.75)	1.7 (4.16)	0 (0)	152.27 (32.6)	12.92 (4.93)	0 (0)	
Feta	Cow				49.00	77.00	60.00	70.00	0.00	2.00	5.00	0.00	[139]
Karish					145.00	160.00	80.00	230.00	0.00	8.00	12.00	0.00	
Mozzarella					140.00	159.00	165.00	290.00	0.00	7.80	16.00	0.00	
Rumi					125.00	520.00	100.00	400.00	0.00	10.00	17.00	0.00	
Mish					230.00	900.00	140.00	590.00	0.00	9.00	16.00	0.00	
Goat cheese	Goat	Raw	no starter culture	150	12 (11.31)	30 (4.24)	10 (7.07)	18.5 (13.44)	0 (0)	0 (0)	0 (0)	34 (22.63)	[149]
			starter cultures		22 (9.9)	4.5 (0.71)	2.5 (0.71)	1 (1.41)	0 (0)	0 (0)	0 (0)	1 (1.41)	
Cheese	Cow	Raw	short ripening time		0.00	0.00	0.00	0.00	0.00	0.00	0.00	0.00	[68]
	Goat		short ripening time		110.00	63.94	38.75	38.90	0.00	0.00	0.00	0.00	
	Ewe		short ripening time		102.00	233.33	10.40	48.40	0.00	0.00	0.00	48.40	
	Mix		short ripening time		0.00	0.00	0.00	0.00	0.00	0.00	0.00	0.00	
	Cow	Past.	short ripening time		0.00	4.40	0.00	0.00	0.00	0.00	0.00	0.00	
	Goat		short ripening time		0.00	0.00	0.00	0.00	0.00	0.00	0.00	0.00	
	Ewe		short ripening time		60.00	0.00	0.00	0.00	0.00	0.00	0.00	0.00	
	Mix		short ripening time		20.00	0.00	0.00	0.00	0.00	0.00	0.00	0.00	
	Cow	Raw	long ripening time		22.84	67.66	3.27	10.34	0.00	12.40	0.00	4.81	
	Goat		long ripening time		171.30	152.60	160.99	44.81	0.00	0.00	0.00	0.00	
	Ewe		long ripening time		49.84	132.66	138.36	0.00	0.00	0.00	0.00	0.00	
	Mix		long ripening time		65.18	216.85	127.03	85.80	0.00	5.70	0.00	0.00	
	Cow	Past.	long ripening time		21.80	26.96	97.68	194.20	0.00	2.20	0.00	0.00	
	Goat		long ripening time		13.84	15.24	58.46	0.00	0.00	0.00	0.00	0.00	
	Ewe		long ripening time		0.00	150.53	9.06	0.00	0.00	0.00	0.00	0.00	
	Mix		long ripening time		10.62	4.34	3.31	0.00	0.00	0.00	0.00	0.00	
Blue cheese	Cow	Raw			210.00	188.82	15.40	137.63	0.00	0.00	0.00	0.00	
	Mix				462.00	508.89	236.07	320.85	0.00	0.00	0.00	27.42	
		Past.			56.51	117.16	46.74	61.15	0.00	0.00	0.00	0.00	
					253.87	229.59	95.25	173.57	0.00	0.00	0.00	2.10	
Gouda	Cow	Past.	starter cultures (*E. faecalis*; *E. faecium*)	0	0.00	0.00	0.00	0.00	0.00	0.00	0.00	0.00	[150]
				14	0.00	56.03	0.00	0.00	0.00	0.00	0.00	0.00	
				28	0.00	122.85	0.00	0.00	0.00	0.00	0.00	0.00	
				42	0.00	170.83	0.00	0.00	0.00	0.00	0.00	0.00	
				56	0.00	233.96	2.28	0.00	0.00	0.00	0.00	0.00	
				70	0.00	242.27	1.54	0.00	0.00	0.00	0.00	0.00	
				84	0.00	476.82	1.93	0.00	0.00	0.00	0.00	0.00	

The numbers in brackets refer to the standard deviations calculated when the dataset contains replicates and not average content. Abbreviations: His (histamine), Tyr (tyramine), Put (putrescine), Cad (cadaverine), Try (tryptamine), Sme (spermine), Spd (spermidine), Phe (phenylethylamine).

## Data Availability

No new data were created or analyzed in this study. Data sharing is not applicable to this article.

## Supplementary Materials

The following supporting information can be downloaded at: https://www.mdpi.com/article/10.3390/foods13162583/s1, Deepening S1. References [194,195,196,197,198,199,200,201,202,203,204,205,206,207] are cited in the Supplementary Materials.
