# Supplementary material for: A Comprehensive Review on the Biogenic Amines in Cheeses: Their Origin, Chemical Characteristics, Hazard and Reduction Strategies
Source: Foods. 2024 Aug 18;13(16):2583. doi: 10.3390/foods13162583 (PMC11353796; doi:10.3390/foods13162583)
Supplement: Supplementary file 1 [file foods-13-02583-s001.zip › foods-3138260-supplementary.pdf]

Biogenic amines could be responsible of foodborne illness; therefore, it is important to limit their formation in foods for two reasons:

i) one of the characteristics that makes these molecules dangerous is their stability to any kind of treatment (heating, freezing, smoking and others) [194];

ii) even little formation could be a hazard for consumers considering how the toxic effect varies according to the consumer sensitivity, with some people showing symptoms with few ppm.

Independently by the concentration of BAs, their intake leads to several health issues with acute or chronic consequences. Among the former, it is possible to mention hypotension, urticaria, flushing, oral burning, headache, abdominal cramps, diarrhoea, vomiting. By contrast, chronic illness can include the onset of immuno-pathologies, Parkinson's and Alzheimer's diseases, and depression [35, 69, 195, 196].

Histamine is one of the most studied, being responsible for several outbreaks of poisoning in fish and cheeses causing hypotension, headache, abdominal cramps, diarrhoea, and vomiting [37]. Its toxic effect is dose-dependent, influenced both by the presence of other BAs or the activity of the oxidase enzymes in the consumer [20]. Tyramine and phenylethylamine cause hypertensive crises and headaches, while putrescine and cadaverine promote the formation of nitrosamines, which are reasonably related to cancer. Finally, tryptamine is responsible of blood pressure increasing and consequently hypertension [24, 54, 197, 198]. A synergistic effect was observed among different BAs, for example, cadaverine and putrescine interfere with the human detoxifying enzymes activity of the BAs, increasing the poisoning risk of tyramine and histamine [35].

Anyway, avoiding poisoned foods is difficult, as their presence is not always detectable by human senses (i.e., food with apparent good organoleptic properties could contain high amounts of histamine or tyramine), and consumers are unconsciously exposed to high levels of BAs, causing food-borne issues. Since their presence in food represent an important hazard, it is important to know the amount of the BAs present in foods. Moreover, taking into consideration how our diet is heterogeneous, the BAs total intake is given by the sum of the amine provided by the foods that make it up. BAs in foods could be an index of the quality of the

product, as they can also cause cancer through their reaction with some food preservatives (as nitrite salts) to form nitrosamines [195].

Relating the BAs content to symptoms is not enough, as it depends on the ability of the consumer to detoxify these molecules, its sensitiveness, and other factors including ongoing comorbidities. To date, there is no maximum legal limit of allowable amines in a food – except for fishery products associated to high histidine accumulation species, and fishery products which have undergone enzyme maturation treatment in brine (histamine concentration <200 and 400 mg/kg, respectively) – as current information on BAs is insufficient for establishing the No Observable Adverse Effect (NOAEL) and Acute Reference Dose (ARfD). Thus, to deepen the topic, a survey was conducted by European Union in 2011. Among considered foods, the dairy products were also monitored, and the output showed that 85% of cheeses studied contained histamine, 75% tyramine, while putrescine, cadaverine, and tryptamine were found very frequently although with a lower incidence [30, 37]. Noteworthy, histamine and tyramine are considered the most dangerous BAs due to their high toxicity, which has led to suggest threshold values by EFSA. Limited information on histamine in both healthy and sensitive individuals has led to the establishment of a NOAEL of 50 mg *per* person per meal for healthy consumers. For individuals with histamine intolerance, values below the detectable limits are considered safe. Concerning tyramine, information is insufficient to establish the NOAEL in humans, but a concentration between 100 and 800 ppm of product is considered acceptable for fermented foods [199]. However, the BIOHAZ panel of EFSA indicated an exposure to 600 mg/person/meal as safe for healthy individuals, whereas lower concentrations were indicated for monoamine oxidase inhibitor drugs consumers (50 and 6 mg for third generation and classical drugs respectively). For putrescine and cadaverine, there is not enough information about their toxicological doses or contribute to enhancing the toxicity of other BAs [37]. Since there is no official regulation for all countries, some of them have imposed some limiting standards for certain BAs, for example: Turkey has set a limit of 10 ppm of histidine in wines [200], Netherland and Czech Republic have set an upper limit of 200 ppm of histidine for meat products, Slovak Republic has an histamine limit of 20 ppm in beers and 200 ppm of tyramine in cheeses [201]. Based on the available literature, some authors tried to quantify and define the range or maximum level acceptable in dairy

products. Santos [20] suggested a maximum total BAs limit of 790 to 900 ppm, values above 1000 ppm represent a hazard, whereas Arlorio et al. [65] reported a total content of 500 mg/kg of BAs as cause of the "cheese reaction", while 200 mg/kg could be a hazard concentration for consumers assuming the amine oxidase inhibitor. Concerning the single BAs: Karovičová and Kohajdová [201] reported that 100 ppm of histamine provides for moderate toxic effects, while values above 1000 ppm could be considered very dangerous, moreover, 5-10 ppm of histamine can be considered hazardous for vulnerable consumers. Rauscher-Gabernig et al. [202] after a survey on histamine concentration in Austrian foods and consumer diet habits, proposed a histamine safe threshold of 400 mg/kg of product, whereas, reasonably speaking, values above 1000 mg could cause severe symptoms [33]. Tyramine toxic threshold has been defined by monitoring the increase in systolic blood pressure (SBP) of healthy consumers, concluding that a dose ranged from 600 to 2000 mg cause an increase in the SBP [203-205]. Patat et al. [206] stated that a concentration of 1100 mg of tyramine causes an increase of SBP of 30 mmHg in 50% of the healthy individuals analysed. Finally, EFSA [37] and Nout [199] stated that an intake of 600 mg per meal had no adverse effect, and values ranging between 100 and 800 mg/kg are reasonably accepted. Rauscher-Gabernig et al. [207] tried to determine the acceptable levels of cadaverine and putrescine in cheese based on the consumption pattern in Austria, based on the intake per person of such molecules via cheese, they have suggested a maximum tolerable levels of 540 and 180 mg/kg of cheese for cadaverine and putrescine respectively.
